# Supplementary material for: Post-operative patient-related risk factors for chronic pain after total knee replacement: a systematic review
Source: BMJ Open. 2017 Nov 3;7(11):e018105. doi: 10.1136/bmjopen-2017-018105 (PMC5695416; doi:10.1136/bmjopen-2017-018105)
Supplement: Appendix 2 [file bmjopen-2017-018105supp002.pdf]

# Appendix 2: Search terms

**MEDLINE (Ovid) (1946 to 17 October 2016)**

1 Epidemiologic Studies/

2 exp Case-Control Studies/

3 exp Cohort Studies/

4 Cross-Sectional Studies/

5 (epidemiologic adj (study or studies)).ab,ti.

6 case control.ab,ti.

7 (cohort adj (study or studies)).ab,ti.

8 cross sectional.ab,ti.

9 cohort analy$.ab,ti.

10 (follow up adj (study or studies)).ab,ti.

11 longitudinal.ab,ti.

12 retrospective$.ab,ti.

13 prospective$.ab,ti.

14 (observ$ adj3 (study or studies)).ab,ti.

15 exp clinical study/

16 randomized controlled trial/

17 15 not 16

18 adverse effect?.ab,ti.

19 1 or 2 or 3 or 4 or 5 or 6 or 7 or 8 or 9 or 10 or 11 or 12 or 13 or 14 or 17 or 18

20 Arthroplasty, Replacement, Knee/

21 Knee Prosthesis/

22 (arthoplast$ adj3 knee$).mp. [mp=title, abstract, original title, name of substance word, subject heading word, keyword heading word, protocol supplementary concept word, rare disease supplementary concept word, unique identifier]

23 (knee$ adj3 replac$).mp. [mp=title, abstract, original title, name of substance word, subject heading word, keyword heading word, protocol supplementary concept word, rare disease supplementary concept word, unique identifier]

24 (knee adj3 implant$).mp. [mp=title, abstract, original title, name of substance word, subject heading word, keyword heading word, protocol supplementary concept word, rare disease supplementary concept word, unique identifier]

25 20 or 21 or 22 or 23 or 24

26 19 and 25

**EMBASE (Ovid) (1980 to 17 October 2016)**

1 Epidemiologic Studies/

2 exp Case-Control Studies/

3 exp Cohort Studies/

4 Cross-Sectional Studies/

5 (epidemiologic adj (study or studies)).ab,ti.

6 case control.ab,ti.

7 (cohort adj (study or studies)).ab,ti.

8 cross sectional.ab,ti.

9 cohort analy$.ab,ti.

10 (follow up adj (study or studies)).ab,ti.

11 longitudinal.ab,ti.

12 retrospective$.ab,ti.

13 prospective$.ab,ti.

14 (observ$ adj3 (study or studies)).ab,ti.

15 exp clinical study/

16 randomized controlled trial/

17 15 not 16

18 adverse effect?.ab,ti.

19 1 or 2 or 3 or 4 or 5 or 6 or 7 or 8 or 9 or 10 or 11 or 12 or 13 or 14 or 17 or 18

20 Arthroplasty, Replacement, Knee/

21 Knee Prosthesis/

22 (arthoplast$ adj3 knee$).mp. [mp=title, abstract, original title, name of substance word, subject heading word, keyword heading word, protocol supplementary concept word, rare disease supplementary concept word, unique identifier]

23	(knee$ adj3 replac$).mp. [mp=title, abstract, original title, name of substance word, subject heading word, keyword heading word, protocol supplementary concept word, rare disease supplementary concept word, unique identifier]

24	(knee adj3 implant$).mp. [mp=title, abstract, original title, name of substance word, subject heading word, keyword heading word, protocol supplementary concept word, rare disease supplementary concept word, unique identifier]

25	20 or 21 or 22 or 23 or 24

26	19 and 25

**PsycINFO (inception [1806] to 23 March 2016**

1. (knee$ adj3 arthoplast$).mp. [mp=title, abstract, heading word, table of contents, key concepts, original title, tests & measures]

2. (knee$ adj3 replac$).mp. [mp=title, abstract, heading word, table of contents, key concepts, original title, tests & measures]

3. (knee$ adj3 surg$).mp. [mp=title, abstract, heading word, table of contents, key concepts, original title, tests & measures]

4. (knee$ adj3 implant$).mp. [mp=title, abstract, heading word, table of contents, key concepts, original title, tests & measures]

5. (knee adj3 prosthe$).mp. [mp=title, abstract, heading word, table of contents, key concepts, original title, tests & measures]

6. 1 or 2 or 3 or 4 or 5
