# Supplementary material for: Post-operative patient-related risk factors for chronic pain after total knee replacement: a systematic review
Source: BMJ Open. 2017 Nov 3;7(11):e018105. doi: 10.1136/bmjopen-2017-018105 (PMC5695416; doi:10.1136/bmjopen-2017-018105)
Supplement: Appendix 3 [file bmjopen-2017-018105supp003.pdf]

Appendix 3: Ongoing studies

Ongoing (in recruitment or active) studies identified in a search of ClinicalTrials.gov on the 18th August 2017 using search terms of ‘pain’, ‘observational studies’, ‘knee replacement’, and ‘adult, senior’

ClinicalTrials.gov Identifier	Study title	Status	Sponsor	Estimated enrollment	Post-operative risk factor(s)	Pain outcome(s)
NCT01320150	Risk Factors and Mechanisms for Persistent Postsurgical Pain After Total Knee Replacement	Recruiting	Rush University Medical Center	300	Area of secondary mechanical hyperalgesia or hypoalgesia, pain intensity	Numerical Rating Scale at 6 months post-operative
NCT02626533	Persistent Postoperative Pain and Joint Stiffness After Total Knee Arthroplasty Performed for Osteoarthritis	Recruiting	Hospital for Special Surgery, New York	186	Range of motion, pain intensity, KOOS scores, neuropathic pain, time to attainment of inpatient physical therapy goals	Numerical Rating Scale at 6 months post-operative
NCT01390298	Pain and Function After Orthopedic Surgery	Recruiting	Wake Forest University	75	Pain	McGill Pain Questionnaire Short Form at post-operative day 168
NCT02156453	Functional Recovery After Total Knee Arthroplasty	Recruiting	Mahidol University	60	Pain, function	Visual Analogue Scale at 1 year post-operative
NCT02579538	Flexibility of Cognition And Persistent Pain	Ongoing, but not recruiting	Washington University School of Medicine	300	Pain	Pain at 1 year post-operative
