# Supplementary material for: Post-operative patient-related risk factors for chronic pain after total knee replacement: a systematic review
Source: BMJ Open. 2017 Nov 3;7(11):e018105. doi: 10.1136/bmjopen-2017-018105 (PMC5695416; doi:10.1136/bmjopen-2017-018105)
Supplement: Author's manuscript [file bmjopen-2017-018105.draft_revisions.pdf]

BMJ Open is committed to open peer review. As part of this commitment we make the peer review history of every article we publish publicly available.

When an article is published we post the peer reviewers' comments and the authors' responses online. We also post the versions of the paper that were used during peer review. These are the versions that the peer review comments apply to.

The versions of the paper that follow are the versions that were submitted during the peer review process. They are not the versions of record or the final published versions. They should not be cited or distributed as the published version of this manuscript.

BMJ Open is an open access journal and the full, final, typeset and author-corrected version of record of the manuscript is available on our site with no access controls, subscription charges or pay-per-view fees (http://bmjopen.bmj.com).

If you have any questions on BMJ Open's open peer review process please email editorial.bmjopen@bmj.com

BMJ Open

Post-operative patient-related risk factors for chronic pain after total knee replacement: a systematic review

Journal:	*BMJ Open*
Manuscript ID	bmjopen-2017-018105
Article Type:	Research
Date Submitted by the Author:	06-Jun-2017
Complete List of Authors:	Wylde, Vikki; University of Bristol, Musculoskeletal Research Unit<br>Beswick, Andrew; University of Bristol, School of Clinical Sciences<br>Dennis, Jane; University of Bristol, Musculoskeletal Research Unit<br>Gooberman-Hill, Rachael; University of Bristol, School of Clinical Sciences
**Primary Subject Heading**:	Surgery
Secondary Subject Heading:	Health services research
Keywords:	Knee < ORTHOPAEDIC & TRAUMA SURGERY, systematic review, Chronic post-surgical pain, Risk factors

SCHOLARONE™ Manuscripts

For peer review only

Post-operative patient-related risk factors for chronic pain after total knee replacement: a systematic review

Wylde V, Beswick AD, Dennis J, Gooberman-Hill R

Musculoskeletal Research Unit, School of Clinical Sciences, University of Bristol, UK

E-mail addresses

Vikki Wylde: v.wylde@bristol.ac.uk

Andrew Beswick: andy.beswick@bristol.ac.uk

Jane Dennis: jane.dennis@bristol.ac.uk

Rachael Gooberman-Hill: r.gooberman-hill@bristol.ac.uk

Corresponding author

Vikki Wylde

Musculoskeletal Research Unit, Learning and Research Building, Southmead Hospital, Bristol, BS10 5NB, UK. Telephone: 0117 414 7878; E-mail: V.Wylde@bristol.ac.uk

Running title: Risk factors for chronic pain after TKR

For peer review only - http://bmjopen.bmj.com/site/about/guidelines.xhtml

ABSTRACT

Objective

To identify post-operative patient-related risk factors for chronic pain after total knee replacement (TKR).

Design

The systematic review protocol was registered on PROSPERO (CRD42016041374). MEDLINE, Embase and PsycINFO were searched from inception to October 2016 with no language restrictions. Key articles were also tracked in ISI Web of Science. Cohort studies evaluating the association between patient-related factors in the first three months post-operative and pain at six months or longer after primary TKR surgery were included. Screening, data extraction and assessment of methodological quality were undertaken by two reviewers. The primary outcome was pain severity in the replaced knee measured with a patient-reported outcome measure at six months or longer after TKR. Secondary outcomes included adverse events and other aspects of pain recommended by the core outcome set for chronic pain after TKR.

Results

After removal of duplicates, 16,430 articles were screened, of which 805 were considered potentially relevant. After detailed evaluation of full-text articles, 14 studies with data from 1,168 participants were included. Post-operative patient-related factors included acute pain (eight studies), function (five studies) and psychosocial factors (four studies). The included studies had diverse methods for assessment of potential risk factors and outcomes and therefore narrative synthesis was conducted. For all post-operative factors, there was insufficient evidence to draw firm conclusions about the association with chronic pain after TKR. Selection bias was a potential risk for all studies, as none were reported to be conducted at multiple centers.

Conclusion

This systematic review found insufficient evidence to draw firm conclusions about the association between any post-operative patient-related factor and chronic pain after TKR. Further high-quality research is required to provide a robust evidence base on post-operative

For peer review only - http://bmjopen.bmj.com/site/about/guidelines.xhtml

risk factors, and inform the development and evaluation of targeted interventions to optimize patients' outcomes after TKR.

Key words: Total knee replacement, post-operative risk factors, chronic pain, systematic review

Strengths and limitations of this study

- This is the first systematic review of patient-related risk factors for chronic pain after total knee replacement.
- Meta-analysis was not possible due to heterogeneity in the assessment of risk factors and outcomes.
- We did not include studies that used a composite pain and function measure to assess outcome.

For peer review only - http://bmjopen.bmj.com/site/about/guidelines.xhtml

INTRODUCTION

Primary total knee replacement (TKR) is a common operation, with over 100,000 operations performed in the UK in 2015 [1, 2] and demand projected to increase dramatically [3]. Patients choose to have a TKR to relieve chronic pain and improve functional ability [4] but approximately 20% of patients experience chronic post-surgical pain [5, 6] defined as pain present at three months after surgery [7]. The impact of chronic pain after TKR is considerable and patients may struggle to cope and adjust to this pain [8]. Provision of services for patients with chronic pain after TKR are patchy and inconsistent [9], with a lack of explicit access points [10]. A systematic review identified that only one intervention has been evaluated for the management of this condition: a single intraarticular botulinum toxin injection [11].

The identification of risk factors for chronic pain after TKR is a fundamental step in designing interventions to improve patient outcomes. Understanding the relevance of non-modifiable factors, such as sex and ethnicity, can help patients and clinicians work together to make informed decisions about TKR. Although some factors may not be modifiable, others may be amenable to intervention. Identification of modifiable patient-related risk factors is an important element in the development of interventions to improve outcome after TKR. Previous systematic reviews have synthesized the literature on pre-operative risk factors for chronic pain after TKR [12-15]. These reviews have found evidence for a range of modifiable pre-operative patient-related risk factors, including pain intensity, catastrophizing, mental health and co-morbidities. Pre-operative interventions have largely focused on exercise and education and have shown little long-term post-operative benefit [15]. Further interventions specifically targeting pain-related behavior, such as cognitive-behavioral patient education and pain coping skills training, are being evaluated [16, 17].

While the potential value for pre-operatively identifying at risk patients and targeting appropriate interventions is clear, multivariable models have been found to have low predictive power, explaining less than 10% of the variability in chronic pain [18]. An operation itself is an important risk factor for chronic pain [19], and factors relating to the operation and early recovery may be important risk factors. A risk index including pre-surgical variables and acute post-surgical pain had “fair” predictive power for the

For peer review only - http://bmjopen.bmj.com/site/about/guidelines.xhtml

development of chronic post-surgical pain across diverse surgery types [20]. Therefore, in addition to evaluating pre-operative risk factors, it is important to consider post-surgical factors that may limit rehabilitation and recovery, and be associated with chronic pain. If patients at risk of developing chronic pain could be identified in the early post-operative period, targeted interventions could be delivered, potentially as part of a comprehensive peri-operative care package, to prevent the development of chronic pain. Although trials evaluating the effectiveness of early post-operative interventions on reducing chronic pain have been conducted [21-24], no systematic review has yet evaluated post-operative risk factors for chronic pain after TKR. Therefore, the aim of this systematic review was to identify early post-operative patient-related risk factors for chronic pain after TKR.

METHODS

Protocol and registration

The protocol was registered on the international prospective register of systematic reviews (PROSPERO) on 6th July 2016 (reference: CRD42016041374). Conduct and reporting of this systematic review adheres to recommendations from PRISMA [25](Appendix 1).

Eligibility criteria

Studies were eligible for inclusion in the review if they met the following PICOS criteria:

Population: Adults undergoing primary TKR predominantly for osteoarthritis. Studies that included TKR patients combined with patients undergoing other orthopaedic procedures were included if separate results were available for TKR patients.

Exposure: Post-operative patient-related risk factors measured in the first three months after surgery. Patients with exposure were those with a risk factor (categorical variable) or higher level of risk factor (continuous variable). The focus of this review was on patient-related risk factors with the potential for modification or use in targeting care, and therefore studies which assessed clinical risk factors such as length of stay, post-operative complications, or radiographic measurements were excluded.

For peer review only - http://bmjopen.bmj.com/site/about/guidelines.xhtml

Comparator: Patients with absence of risk factor (categorical variable) or lower level of risk factor (continuous variable).

Outcome: Severity of pain in the replaced knee measured with a patient-reported outcome measure at six months or longer after TKR surgery.

Study design: Cohort studies that have explored the relationships between factors measured in the first three months post-operative and longer-term pain outcomes.

Information sources and searches

MEDLINE, Embase and PsycINFO were searched from inception to 17th October 2016. Searches were conducted by experienced systematic reviewers (AB and JD) based on established design filters [26, 27]. The search strategy combined terms relating to study design (e.g. cohort, epidemiological study) and population (e.g. knee replacement, knee arthroplasty). Full search strategies are provided in Appendix 2. No language restrictions were applied. Searches were supplemented with hand searching of reference lists and review articles, and key articles were tracked in ISI Web of Science. Conference abstracts were excluded.

Study selection and data extraction

Bibliographic details of the articles identified were exported into EndNote X7 (Thomson Reuters) and duplicates removed. After an initial screening of titles and abstracts by one reviewer (AB) to remove clearly irrelevant studies, titles and abstracts were screened in duplicate by two reviewers (AB and VW). As recommended in the Cochrane Handbook [28], reviewers were 'over inclusive' at early stages and retained any potentially relevant studies. Full text of all such reports were acquired and assessed for eligibility against the PICOS criteria in duplicate by two reviewers (AB and VW). Discrepancies were resolved in discussion with a third reviewer (JD). Data from articles that met the eligibility criteria were extracted into an Excel database by one reviewer (VW) with checking against source articles by a second reviewer (AB or JD). Extracted data comprised: country, date, setting, population, participant demographics, study methodology including statistical analysis, risk factors, time to follow up, losses to follow up, joint-specific pain outcomes, variables

For peer review only - http://bmjopen.bmj.com/site/about/guidelines.xhtml

included in multivariable analyses and information relevant to assessment of study methodological quality.

Where necessary, authors of studies were contacted for further information to enable judgements about eligibility and/or to provide unpublished outcome data relevant to the review. If data from patients with TKR were combined with patients undergoing other orthopaedic procedures, separate data for patients with TKR were requested. If a combined pain and function outcome was reported, such as the Oxford Knee Score or WOMAC score, separate pain-specific data were requested, e.g. the Oxford Knee Score pain subscale or WOMAC pain scale.

Outcomes

The primary outcome was pain severity in the replaced knee measured with a patient-reported outcome measure at six months or longer after TKR. Secondary outcomes included adverse events and other aspects of pain recommended by the core outcome set for chronic pain after TKR [29]. These included pain interference with daily living, pain and physical functioning, temporal aspects of pain, pain description, emotional aspects of pain, use of pain medication, and satisfaction with pain relief. No limits were placed on the tools used to measure these outcomes.

Assessment of methodological quality of included studies

A non-summative checklist, which consisted of four items to assess selection bias (inclusion of consecutive patients and representativeness), bias due to missing data (follow-up rates) and bias due to inadequate consideration of confounding (multivariable or univariable analysis) was developed for use in this review. These items were informed by existing tools, including the MINORS [30], Newcastle-Ottawa quality assessment scale [31] and the ROBINS-I tool [32]. Each item was rated as adequate, not adequate or not reported. Each individual item rating is reported, rather than an overall score, as summative scales risk rating reporting rather than conduct [33]. Ratings of methodological quality for included studies were conducted independently by two reviewers (VW and JD) and any discrepancies were resolved in discussion with a third reviewer (AB).

For peer review only - http://bmjopen.bmj.com/site/about/guidelines.xhtml

Data synthesis

In the protocol, meta-analyses were planned if two or more studies assessed the same risk factor with suitable methodology. In comparing groups of patients with or without a risk factor, outcomes adjusted for baseline patient factors would be considered in preference to unadjusted outcomes and the effect of non-adjustment would be explored in a sub-group analysis. If studies reported categorical pain outcomes, risk ratios would be used to summarize cohort studies and odds ratios for case-control studies. For risk factors reported as continuous variables, results of meta-analyses would be reported as mean differences or standardized mean differences, depending on the consistency of risk factor and outcome measures reported. We planned to explore the effect of non-adjustment for other variables in a sub-group analysis. Assessment of heterogeneity was planned using the chi-squared and I-squared statistic. The protocol stated that we would conduct a sensitivity analyses on methodological quality assessment.

At analysis stage, opportunities for meta-analysis were limited by heterogeneity in the assessment of risk factors and outcomes. Therefore, we undertook a descriptive narrative analysis, in keeping with the approach recommended by the Cochrane Handbook [28].

RESULTS

After removal of duplicates, 16,430 articles were screened, of which 857 were considered potentially relevant. After detailed evaluation of full-text articles, 14 studies with data from 1,613 participants were included [34-47](Figure 1). The most common reasons for excluding potentially relevant studies were because patient-related factors were not assessed and follow-up after TKR surgery was less than six months.

Authors of 17 studies were contacted to clarify eligibility criteria (n=6), request disaggregated data for patients with TKR (n=9) or request pain-specific outcome data (n=2). Disaggregated data for patients with TKR were provided by authors for two studies [41, 47].

Study characteristics

An overview of study characteristics is provided in Table 1. Of the 14 included studies, three were from the UK, two each from Australia, USA and Spain, and one study from Belgium,

For peer review only - http://bmjopen.bmj.com/site/about/guidelines.xhtml

For peer review only

Denmark, France, Portugal and Serbia. Thirteen studies were conducted at a single center and one study did not report the number of centres. Eleven of the studies were cohort studies, two were randomized controlled trials analyzed as cohort studies and one was a case-control study with prospective data collection. Sample sizes ranged from 23 to 402, with a median of 115 participants. One study included a small number of patients undergoing unicompartmental knee replacement but was included in the review as 83% of participants had TKR [45]. Follow-up assessments varied: four studies assessed outcomes at six months after TKR, five at 12 months and the remainder between 3-7 years post-operative. Pain at follow-up was evaluated using the WOMAC Pain scale [48](five studies), numerical rating scale (three studies), visual analogue scale (two studies), American Knee Society Score Pain question [49] (two studies), and verbal descriptor scale (two studies). Secondary outcomes for the review relating to serious adverse events and other aspects of pain outcomes were infrequently reported and therefore not summarized.

Assessment of methodological quality of included studies

Ratings of methodological quality for the 14 included studies are provided in Table 2. Eight studies reported that consecutive patients were recruited, eight studies followed up >80% participants, and nine studies conducted multivariable analysis. All studies had issues relating to selection bias because none were reported as being conducted at multiple centers.

Patient-related post-operative risk factors

Patient-related post-operative risk factors were categorized into three groups: acute post-operative knee pain, knee function and psychosocial factors.

Acute post-operative knee pain

Eight studies including data from 737 participants evaluated the association between pain in the first three months after TKR and chronic pain (Table 3). Timing of acute postoperative pain was classified as: pain within the first postoperative week; pain between one and two weeks postoperative; and pain from two weeks to three months. Pain as a risk factor was assessed using a Visual Analogue Scale (three studies), verbal descriptor scale (two studies), Numeric Rating Scale (two studies), WOMAC Pain scale (one study) and PainDETECT (one

For peer review only - http://bmjopen.bmj.com/site/about/guidelines.xhtml

study). Five studies conducted multivariable analysis, two studies conducted univariable analysis and for one study no statistical analysis was performed as data were provided by authors on a small subset of patients with TKR.

Pain severity on post-operative days 1-7

Four studies with data from 491 participants evaluated whether pain severity in the first week after surgery was associated with chronic pain [37, 41, 43, 45]. Two were at risk of bias due to missing data and one study was at risk of bias due to inadequate consideration of confounding. Methods used to assess pain were verbal descriptor scale [37], VAS [43] and NRS [41, 45], Three studies found that more severe acute post-operative pain was associated with more severe pain at 6-12 months after TKR [37, 43, 45], although in one study this association was attenuated completely after adjustment for pre-operative pain [43]. One study with found no association between pain at 42 hours after surgery and the presence of chronic pain at 4-6 months [41].

Pain severity in post-operative days 8-14

Three studies with data from 191 participants evaluated whether pain severity on post-operative days 8-14 was associated with chronic pain [34, 37, 47]. One study was at risk of bias due to missing data and two studies were at risk of bias due inadequate consideration of confounding. Pain was assessed in two studies with a verbal descriptor scale [37, 47] and in one with the WOMAC pain scale and VAS [34]. Pain on post-operative day eight and at two weeks was not found to be associated with chronic pain in two studies [34, 37], and descriptive data only were available for the study that evaluated pain on post-operative day 10 [47]. In the study with low risk of bias apart from with regard to representativeness [34], pain severity at two weeks was not found to be associated with pain at six months after TKR

Pain severity between 2 weeks and 3 months post-operative

Five studies with data from 314 participants evaluated whether pain severity between two weeks and three months post-operative was associated with chronic pain after TKR [34, 35, 37, 40, 47]. Two studies were at risk of bias due to missing data and three studies were at risk of bias due to inadequate consideration of confounding. Methods to assess pain were the WOMAC pain scale [34], VAS [34, 35, 40] and verbal descriptor scale [37, 47]. In one study with risk of bias associated only with conduct at a single center, pain severity at eight weeks post-operative was found to be associated with pain at six months post-operative when

For peer review only - http://bmjopen.bmj.com/site/about/guidelines.xhtml

assessed with the WOMAC but not the VAS [34]. In one study with univariable analysis, pain severity assessed on day 30 was found to be associated with pain severity at six months but not 12 months after TKR [37]. The same study found that pain at three months post-operative was not associated with pain severity at six months and 12 months post-operative [37]. In another study, neuropathic pain at six weeks post-operative was found to be moderately associated with pain at 39-51 months after surgery [40]. In one study, there was no difference in verbal descriptor scale pain at 12 months in patients with different average pain levels at six weeks [47]. However considering 'worst' pain, 7/14 patients with moderate to severe pain at six weeks reported moderate to severe pain at 12 months compared with 1/9 patients with none or mild pain at six weeks. A study which assessed global pain and night pain at one month and three months post-operative found that they were associated with global pain and night pain respectively at a future time point (six months and 12 months) [35].

Knee function

Five studies including data from 835 participants evaluated the association between post-operative knee function and chronic pain after TKR (Table 4). Three studies were at risk of bias due to missing data and one study was at risk of bias due to inadequate consideration of confounding. Assessment of knee function varied and included range of motion, ambulatory status, WOMAC Function, six minute walk test and stair ascent speed.

Four studies including data from 735 participants evaluated whether function at hospital discharge was associated with chronic pain after TKR [36, 38, 39, 42]. Two of these studies assessed range of motion [36, 42] and two assessed ambulatory status at discharge [38, 39]; none found an association. One study at low risk of bias except inclusion of a single center with 100 patients evaluated whether function at two weeks and eight weeks, assessed using three different methods, was associated with WOMAC Pain scores at six months post-operative [34]. This study found that WOMAC Function score at two weeks, but not eight weeks, was associated with chronic pain; six minute walk test at both two weeks and eight weeks was associated with chronic pain; stair ascent speed at two and eight weeks was not associated with chronic pain.

Psychosocial factors

For peer review only - http://bmjopen.bmj.com/site/about/guidelines.xhtml

Four studies including data from 226 participants evaluated the association between post-operative psychological factors and chronic pain after TKR (Table 5). Two studies were at risk of bias due to missing data and one study was at risk of bias due to inadequate consideration of confounding. Risk factors assessed included catastrophizing, depression, social support, coping skills, fear of movement and anxiety. In one study, catastrophizing at a previous time point was a risk factor for night pain, but not global pain, at a future time point [35]. In the same study, depression was found to be a risk factor for global pain but not night pain. Another study assessing risk factors at six weeks post-operative found that perceived positive social support was associated with less chronic pain, negative social support with more chronic pain, and no association between coping and pain at six months after TKR [44]. At two weeks post-operative, patients with a high fear of movement at two weeks post-operative reported more pain at six months than those with a low fear of movement [46]. Greater anxiety at 48 hours after surgery was found to be associated with a higher risk of having a pain score of >3 on a numeric rating scale at 4-6 months after TKR [41].

DISCUSSION

This is the first systematic review to evaluate post-operative patient-related risk factors for chronic pain after TKR. Fourteen cohort studies were identified which evaluated the association between patient-related factors measured in the first three months post-operative and pain severity measured with a patient-reported outcome measure at six months or longer after primary TKR. Post-operative factors assessed included pain (eight studies), function (five studies) and psychosocial factors (four studies).

For all post-operative patient-related factors, there was insufficient evidence to draw firm conclusions on the association with chronic pain after TKR. When reviewing observational cohort studies, it is essential to consider issues that may introduce bias and lead to potentially misleading results and their interpretation. The key issues relate to generalizability, incomplete follow up and accounting for confounding factors. Regarding generalizability, findings from single-center and multi-center studies can differ [50], and one potential factor contributing to this difference is the recruitment of a more homogeneous population in single-center studies. The population may be highly selected and therefore have limited validity external to the study setting. Losses to follow represent another cause of bias as patients who do not complete longer-term assessments may have poorer outcomes [51, 52]. In this review, six studies had data on <80% participants at follow-up. The methodological quality of five

For peer review only - http://bmjopen.bmj.com/site/about/guidelines.xhtml

studies was limited by the lack of multivariable analysis to minimize the impact of potential confounding on results. In studies with no risk of bias other than patient selection, there was a suggestion that chronic pain was associated with increased acute post-operative pain during the hospital stay [43, 45]. However, in one of these studies, a comprehensive assessment of pain relationships over time suggested that the association was largely explained by pre-operative pain [43]. For later pain assessments, one study did not identify consistent associations between post-operative pain and chronic pain [34].

This review has strengths and weakness which should be considered when interpreting the results. While our search terms were broad to identify cohort studies which involved patients with TKR, three studies were identified through methods other than the main searches. This is a recognized issue in identification of observational studies [53] and highlights the importance in bibliographic databases of appropriate indexing and use of keywords. It is possible that studies including general orthopaedic or surgical populations may have included patients with TKR, and these may not have been identified. However, when these studies were identified, we contacted authors and data for patients with TKR were provided for two studies [41, 47]. The primary outcome of interest in this review was pain at six months or longer after TKR, and therefore we did not include studies that used a composite pain and function measure to assess outcome, for example the total Oxford Knee Score [54] or WOMAC [48]. This is because when such composite measures are reported without any separation of pain from function it is not possible to use the scores to assess pain *per se*. Pre-operative risk factors for post-operative pain and functional limitations are different [18, 55], and therefore it is important to assess pain and function as distinct outcomes. Separate pain and function scores can be calculated for the most commonly used patient-reported outcome measures, the WOMAC [56] and the Oxford Knee Score [57], and future studies would benefit from analyzing these outcomes separately. Research on post-operative risk factors is limited by heterogeneity in the assessment of risk factors and outcomes. If greater standardization could be achieved, such as through the implementation of core outcome sets [29], future systematic reviews may be able to pool data in meta-analysis to provide evidence for post-operative patient-related risk factors for chronic pain after TKR.

Much of the research evaluating risk factors for outcomes after TKR has focused on the pre-operative period rather than the period after surgery [12]. Numerous pre-operative patient-related factors and their association to chronic pain have been evaluated, including knee pain severity and duration, pain at other sites, comorbidities, function, depression, social support,

For peer review only - http://bmjopen.bmj.com/site/about/guidelines.xhtml

anxiety, fear of movement, pessimism and quality of life [12]. In comparison, our review found that the current extent of research into post-operative risk factors is narrow, and further research is needed. Assessing potential post-operative risk factors is important as some factors may be more associated with outcome when measured in the post-operative, rather than the pre-operative period [58]. Prediction of chronic post-surgical pain has been found to be strongest when assessing both pre-operative and post-operative risk factors [20]. Factors specific to the post-operative recovery period, such as acute post-operative pain, and factors which span the peri-operative period, such as anxiety, have the potential to influence outcomes. Identification of both pre-operative and post-operative risk factors could inform the development of comprehensive care packages to improve outcomes.

Despite the lack of sufficient evidence about post-operative risk factors, research has evaluated whether early post-operative interventions improve longer-term outcomes after TKR. The long-term effects of pharmacological interventions to reduce pain severity in the early post-operative period have been evaluated, both in patients undergoing TKR and other surgical procedures [21, 22]. While effective at reducing acute post-operative pain, numerous peri-operative pharmacotherapies are not effective at preventing chronic post-surgical pain. Similarly, outpatient physiotherapy interventions to improve early post-operative function have little effect on long-term pain [23, 24]. This may be because acute post-operative pain and functional limitations are not risk factors for chronic pain after TKR or it may be that these interventions require evaluation in trials that are focused on high risk patients. However, before evaluation of such stratified models of care is possible, more research is needed to identify post-operative patient-related risk factors for chronic pain after TKR.

In conclusion, this systematic review found insufficient evidence to draw conclusions about the association between any post-operative patient-related factor and chronic pain after TKR. Further high-quality research is required to provide a robust evidence on post-operative risk factors, and inform the development and evaluation of targeted interventions to optimize patients' outcomes after TKR.

For peer review only

For peer review only - http://bmjopen.bmj.com/site/about/guidelines.xhtml

Acknowledgements

We would like to thank all the study authors who took the time to reply to our requests for further clarification or additional data.

Author contributions

All authors contributed to the concept and design of the study. ADB, JD and VW contributed to the acquisition and analysis of data. VW drafted the article and ADB, JD and RGH revised it critically for important intellectual content. VW and ADB take responsibility for the integrity of the work as a whole, from inception to finished article.

Funding

This article presents independent research funded by the National Institute for Health Research (NIHR) under its Programme Grants for Applied Research programme (RP-PG-0613-20001). The views expressed are those of the authors and not necessarily those of the NHS, the NIHR or the Department of Health. The funder had no role in the study design, collection, analysis and interpretation of data; in the writing of the manuscript; or in the decision to submit the manuscript for publication.

Competing interests

The authors have no conflicts of interest to declare.

Data sharing statement

For peer review only - http://bmjopen.bmj.com/site/about/guidelines.xhtml

For peer review only

No additional data are available.

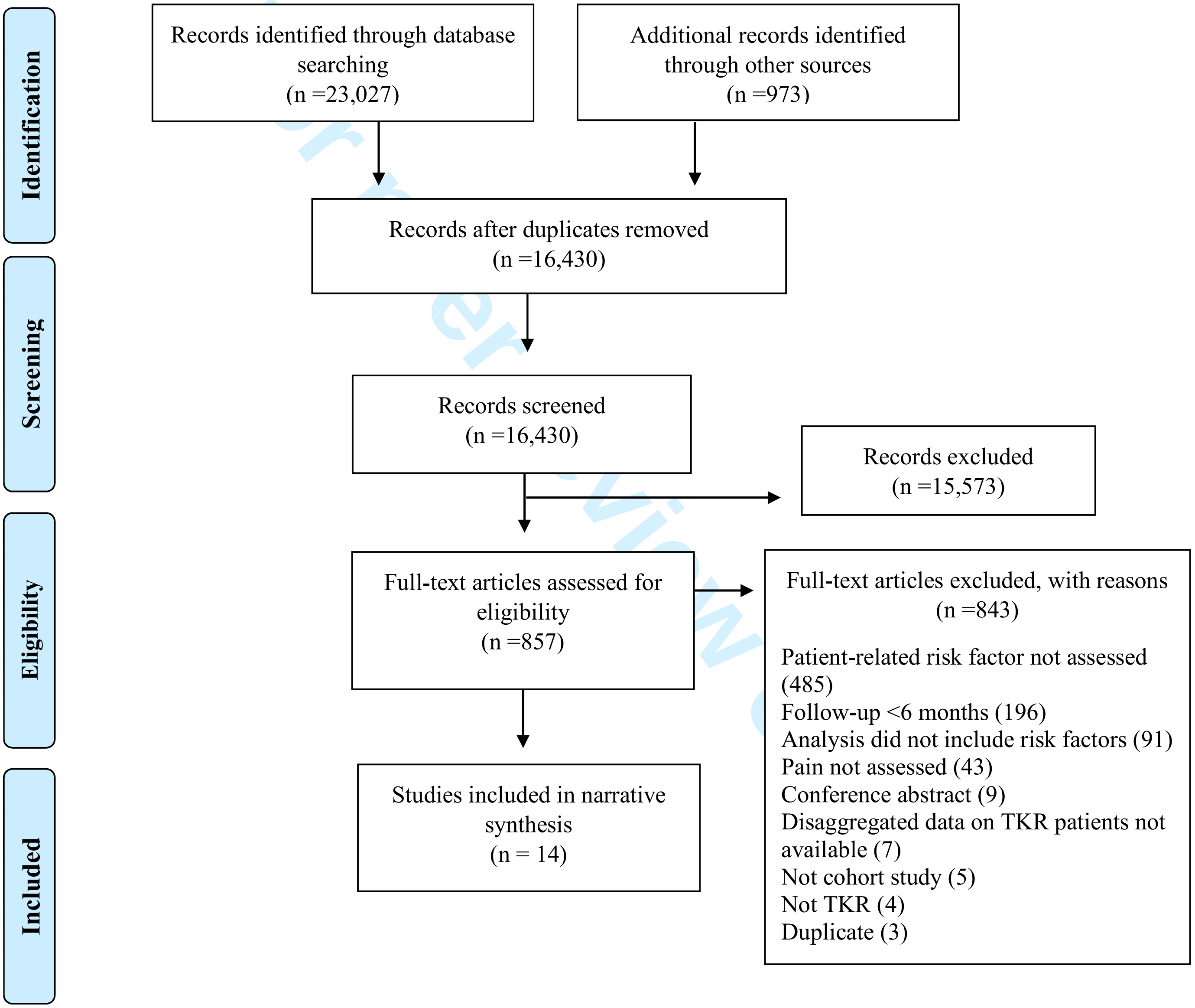

Figure 1: Systematic review flow diagram

For peer review only - http://bmjopen.bmj.com/site/about/guidelines.xhtml

Table 1: Characteristics of included studies

Study	Dates of baseline data collection	Study design	Country	Participants recruited/at final follow-up	Mean/ median age	% female	Outcome measure	Duration of follow-up
Crosbie (2010)[34]	2005-2006	Cohort*	Australia	102/100	68	56%	WOMAC Pain	6 months
Edwards (2009) [35]	Not reported	Cohort	USA	43 in analysis	72	58%	VAS	12 months
Elson (2006) [36]	1995-1998	Case control	UK	622/402 knees	69	54%	AKSS pain question	5 years
Grosu (2016) [37]	2009-2010	Cohort	Belgium	114/68	66	66%	Verbal descriptor scale	12 months
Nunez (2007) [38]	2000-2001	Cohort	Spain	88/67	75	81%	WOMAC Pain	3 years
Nunez (2009) [39]	2000	Cohort	Spain	142/112	67	77%	WOMAC Pain	7 years
Phillips (2014) [40]	2009-2010	Cohort	UK	96/80	71	56%	VAS	39-51 months
Pinto (2013) [41]	2009-2011	Cohort	Portugal	42 in analysis	66	77%	NRS	4-6 months
Riis (2014) [42]	2007-2009	Cohort	Denmark	176 /154	68	65%	AKSS pain question	12 months
Sayers (2016) [43]	2009-2012	Cohort*	UK	316/277	69	53%	WOMAC Pain	12 months
Stephens (2002) [44]	Not reported	Cohort	USA	71/63	67	54%	WOMAC Pain	6 months
Thomazeau (2016) [45]	2013	Cohort	France	109/104	69	72%	NRS	6 months
Kocic (2015) [46]	2007-2013	Cohort	Serbia	78/78	68	76%	NRS	6 months
Veal (2015) [47]	2013	Cohort	Australia	23 in analysis	Not available	Not available	Verbal descriptor scale	12 months

*RCT analysed as cohort

For peer review only

For peer review only - http://bmjopen.bmj.com/site/about/guidelines.xhtml

Table 2: Ratings of methodological quality for included studies

Study	Inclusion of consecutive patients	Representativeness (multi-center adequate)	Percent follow-up (>80% adequate)	Minimization of potential confounding (multivariable analysis adequate)
Crosbie (2010)[34]	+	-	+	+
Edwards (2009) [35]		-	-	+
Elson (2006) [36]		-	-	-
Grosu (2016) [37]		-	-	-
Nunez (2007) [38]	+	-	-	+
Nunez (2009) [39]	+	-	-	+
Phillips (2014) [40]	+	-	+	-
Pinto[1] (2013) [41]	+	-	-	+
Riis (2014) [42]	+	-	+	+
Sayers (2016) [43]	+[2]	-	+	+
Stephens (2002) [44]			+	+
Thomazeau (2016) [45]	+	-	+	+
Kocic (2015) [46]		-	+	-
Veal[1] (2015) [47]		-	+	-

'+' adequate, '-' inadequate, 'blank' not reported

[1]For studies which authors provided data on patients with TKR, ratings are based on the study as reported in the article; [2] Information obtained through personal contact.

For peer review only - http://bmjopen.bmj.com/site/about/guidelines.xhtml

For peer review only

Table 3: Studies evaluating acute post-operative knee pain as a risk factor for chronic pain after TKR

Author and date	Number in analysis	Risk factor measurement	Outcome(s)	Univariable or multivariable analysis	Association	Results summary
Edwards 2009 [35]	43	Global pain VAS at 1 month and 3 months<br><br>Night pain VAS at 1 month and 3 months	Global pain VAS at 6 and 12 months<br><br>Night pain VAS at 6 and 12 months	Multivariable generalized estimating equation model	Yes<br><br>Yes	Global pain at a previous time point was a predictor of global pain at a future time point (estimate=0.43, SE=0.08, t=5.8, p<0.001)<br><br>Night pain at a previous time point was a predictor of night pain at a future time point (estimate=0.32, SE=0.08, t=3.8, p<0.001)
Crosbie 2010 [34]	100	WOMAC Pain Scale at 2 weeks<br><br>VAS at 2 weeks<br><br>WOMAC Pain at 8 weeks<br><br>VAS at 8 weeks	WOMAC Pain scale at 6 months	Multivariable linear regression	No<br><br>No<br><br>Yes<br><br>No	Not significant, results not reported<br><br>Not significant, results not reported<br><br>Beta coefficient = +0.25 ± 0.07<br><br>Not significant, results not reported
Pinto 2013 [41]	42	NRS at 48 hours	NRS at 4-6 months	Hierarchical logistic regression	No	Exp(B) =0.998 (95% CI 0.623-1.601), p value=0.995
Phillips 2014 [40]	80	PainDETECT at 6 weeks	Pain VAS at 39-51 months	Univariable correlation	Yes	PainDETECT at 6 weeks correlated moderately with VAS pain scores (r=0.53)
Veal 2015 [47]	23	Verbal descriptor scale for average pain at 10 days	Verbal descriptor scale for average pain at 12 months	N/A – statistical analysis inappropriate as data provided by authors on a small subset of patients	N/A	11 patients had none/mild pain at 10 days, none of these patients had severe/moderate pain at 12 months.<br><br>12 patients had moderate/severe pain at 10 days, 2 of these patients had moderate/severe at 12 months.

For peer review only - http://bmjopen.bmj.com/site/about/guidelines.xhtml

		Verbal descriptor scale for worst pain at 10 days	Verbal descriptor scale for worse pain at 12 months			2 patients had none/mild pain at 10 days, none of these patients had severe/moderate pain at 12 months.<br><br>21 patients had moderate/severe pain at 10 days, 8 of these patients had moderate/severe at 12 months.
		Verbal descriptor scale for average pain at 6 week	Verbal descriptor scale for average pain at 12 months			17 patients had none/mild pain at 6 weeks, 1 of these patients had moderate/severe pain at 12 months.<br><br>6 patients had moderate/severe pain at 6 weeks, 1 of these patients had moderate/severe at 12 months.
		Verbal descriptor scale for worst pain at 6 weeks	Verbal descriptor scale for worse pain at 12 months			9 patients had none/mild pain at 6 weeks, 1 of these patients had severe/moderate pain at 12 months.<br><br>14 patients had moderate/severe pain at 6 weeks, 7 of these patients had moderate/severe at 12 months.
Grosu 2016 [37]	68	Verbal descriptor scale on days 1,2 and 3 (cumulative value of maximal pain intensity)	Verbal descriptor scale at 6 months	Univariable correlation	Yes	r=0.350; p value = 0.009
			Verbal descriptor scale at 12 months		Yes	r=0.350; p value = 0.009
		Verbal descriptor scale on day 8	Verbal descriptor scale at 6 months		No	Not significant, results not reported
			Verbal descriptor scale at 12 months		No	Not significant, results not reported

For peer review only - http://bmjopen.bmj.com/site/about/guidelines.xhtml

		Verbal descriptor scale on day 30	Verbal descriptor scale at 6 months		Yes	r=0.310, p=0.013
			Verbal descriptor scale at 12 months		No	Not significant, results not reported
		Verbal descriptor scale at 3 months	Verbal descriptor scale at 6 months		No	Not significant, results not reported
			Verbal descriptor scale at 12 months		No	Not significant, results not reported
Sayers 2016 [43]	277	VAS for pain on rest on days 1,2 and 3 (combined)	WOMAC Pain at 12 months	Multivariable structural equation modelling	Yes	Beta=0.222, SE=0.058, 95% CI = 0.109, 0.336, p value = 0.0001<br><br>When pre-operative pain added: Beta=0.09, 95% CI = -0.09, 0.27, p value = 0.332
		VAS for pain on movement on days 1,2 and 3(combined)			Yes	Beta=0.140, SE=0.044, 95% CI = 0.054, 0.226, p value = 0.0014<br><br>When pre-operative pain added: Beta=0.00, 95% CI = -0.14, 0.15, p value = 0.955
Thomazeau 2016 [45]	104	NRS on days 1-4	NRS at 6 months	Multivariate logistic regression	Yes	Patients with high intensity acute post-operative pain (defined though Latent Class Growth analysis) were more likely to have pain at 6 months than patients with low intensity acute post-operative pain (OR=4.23, 95% CI=1.39-12.88, p-value=0.011)

For peer review only

For peer review only - http://bmjopen.bmj.com/site/about/guidelines.xhtml

Table 4: Studies evaluating post-operative knee function as a risk factor for chronic pain after TKR

Author and date	Number in analysis	Risk factor measurement	Outcome	Univariable or multivariable analysis	Association	Results summary
Elson and Brenkel 2006 [36]	402 knees	Range of motion (active and passive) at hospital discharge	AKSS Pain at 5 years	Univariable analysis	No	Not significant, results not reported
Nunez 2007 [38]	67	Ambulatory status at hospital discharge	WOMAC Pain at 3 years	Multivariable linear regression	No	Not significant, results not reported
Nunez 2009 [39]	112	Ambulatory status at hospital discharge	WOMAC Pain at 7 years	Multivariable linear regression	No	Not significant, results not reported
Crosbie 2010 [34]	100	WOMAC Function at 2 weeks<br><br>6 minute walk test at 2 weeks<br><br>Stair ascent speed at 2 weeks<br><br>WOMAC Function at 8 weeks<br><br>6 minute walk test at 8 weeks<br><br>Stair ascent speed at 8 weeks	WOMAC Pain at 6 months	Multivariable linear regression	Yes<br><br>Yes<br><br>No<br><br>No<br><br>Yes<br><br>No	Beta coefficient = +0.06, SE = ± 0.02.<br><br>Beta coefficient = -0.05, SE = ± 0.01.<br><br>Not significant, results not reported<br><br>Not significant, results not reported<br><br>Beta coefficient = -0.04, SE = ± 0.01.<br><br>Not significant, results not reported
Riis 2014 [42]	154	Range of flexion (active) at hospital discharge	AKSS Pain at 12 months	Multivariable binary logistic regression	No	OR 1.00 (95% CI 0.99 to 1.04), p=0.698

For peer review only - http://bmjopen.bmj.com/site/about/guidelines.xhtml

Table 5: Studies evaluating post-operative psychological factors as risk factors for chronic pain after TKR

Author and date	Number in analysis	Risk factor measurement	Outcome(s)	Univariable or multivariable analysis	Association	Results summary
Stephens 2002 [44]	63	Perceived positive social support (MOS Social Support survey) at 6 weeks	WOMAC Pain at 6 months	Multivariable hierarchical multiple regression	Yes	Beta=-0.29, SE=0.09, p≤0.05
		Perceived negative social support (4 items) at 6 weeks			Yes	Beta=-0.27, SE=0.14, p≤0.05
		Active coping (Vanderbilt Multidimensional Pain Coping Inventory Active Coping scale) at 6 weeks			No	Beta=-0.14, SE=0.01
		Avoidant coping (Vanderbilt Multidimensional Pain Coping Inventory Avoidant Coping scale) at 6 weeks			No	Beta=0.21, SE=0.01
Edwards 2009 [35]	43	Catastrophizing (Coping Strategies Questionnaire catastrophizing subscale) at 1 month and 3 months	Global pain VAS at 6 and 12 months	Multivariable generalized estimating equation model	No	Catastrophizing at a previous time point was not a predictor of global pain at a future time point (estimate=2.1, SE=2.2, t=0.9, p=0.35)
			Night pain VAS at 6 and 12 months		Yes	Catastrophizing at a previous time was a predictor of nighttime pain at a future time point (estimate=5.1, SE=2.5, t=2.0, p=0.04).
		Depression (Centre for Epidemiological Studies Depression Scale at 1 month and 3 months)	Global pain VAS at 6 and 12 months		Yes	Depression at a previous time point was a predictor of global pain at a future time point (estimate=0.67, SE=0.30, t=2.2, p=0.03)

For peer review only - http://bmjopen.bmj.com/site/about/guidelines.xhtml

			Night pain VAS at 6 and 12 months		No	Depression at a previous time point was not a predictor of nighttime pain at a future time point (estimate=0.40, SE=0.33, t=1.2, p=0.24).
Pinto 2013 [41]	42	Anxiety scale (Hospital Anxiety and Depression Scale) at 48 hours	NRS at 4-6 months	Hierarchical logistic regression	Yes	Exp(B) = 1.713 (95% CI 1.104-2.657), p value=0.016
Kocic 2015 [46]	78	Fear of movement (Tampa Scale of Kinesiophobia) at 2 weeks	NRS at 6 months	Univariable comparison of means	Yes	Patients with high fear of movement had more pain (mean=3.24, SD=1.98) than patients with low fear of movement (mean=1.81, SD=1.5), p=0.0035

For peer review only - http://bmjopen.bmj.com/site/about/guidelines.xhtml

References

1. National Joint Registry for England W, Northern Ireland and the Isle of Man. 13th Annual Report Hemel Hempstead: NJR Centre.2016.
2. Scottish Arthroplasty Project. Biennial report NHS National Services Scotland2016.
3. Maradit Kremers H, Larson DR, Crowson CS, Kremers WK, Washington RE, Steiner CA, et al. Prevalence of Total Hip and Knee Replacement in the United States. J Bone Joint Surg Am 2015; 97: 1386-1397.
4. Scott CE, Bugler KE, Clement ND, MacDonald D, Howie CR, Biant LC. Patient expectations of arthroplasty of the hip and knee. J Bone Joint Surg Br 2012; 94: 974-981.
5. Beswick AD, Wylde V, Gooberman-Hill R, Blom A, Dieppe P. What proportion of patients report long-term pain after total hip or knee replacement for osteoarthritis? A systematic review of prospective studies in unselected patients. BMJ Open 2012; 2: e000435.
6. Wylde V, Hewlett S, Learmonth ID, Dieppe P. Persistent pain after joint replacement: Prevalence, sensory qualities, and postoperative determinants. Pain 2011; 152: 566-572.
7. Werner MU, Kongsgaard UE. I. Defining persistent post-surgical pain: is an update required? Br J Anaesth 2014; 113: 1-4.
8. Jeffery AE, Wylde V, Blom AW, Horwood JP. "It's there and I'm stuck with it": patients' experiences of chronic pain following total knee replacement surgery. Arthritis Care Res (Hoboken) 2011; 63: 286-292.
9. Wylde V, Mackichan, Dixon S, Gooberman-Hill R. Service provision for patients with chronic post-surgical pain after total knee replacement: An evaluation of current practice. Journal of Pain Management 2014; 7: 147-154.
10. MacKichan F, Wylde V, Gooberman-Hill R. Pathways Through Care for Long-Term Pain After Knee Replacement: A Qualitative Study With Healthcare Professionals. Musculoskeletal Care 2015.
11. Beswick AD, Wylde V, Gooberman-Hill R. Interventions for the prediction and management of chronic postsurgical pain after total knee replacement: systematic review of randomised controlled trials. BMJ Open 2015; 5: e007387.

For peer review only - http://bmjopen.bmj.com/site/about/guidelines.xhtml

12. Lewis GN, Rice DA, McNair PJ, Kluger M. Predictors of persistent pain after total knee arthroplasty: a systematic review and meta-analysis. British Journal of Anaesthesia 2015; 114: 551-561.
13. Baert IA, Lluch E, Mulder T, Nijs J, Noten S, Meeus M. Does pre-surgical central modulation of pain influence outcome after total knee replacement? A systematic review. Osteoarthritis Cartilage 2016; 24: 213-223.
14. Hernandez C, Diaz-Heredia J, Berraquero ML, Crespo P, Loza E, Ruiz Iban MA. Pre-operative Predictive Factors of Post-operative Pain in Patients With Hip or Knee Arthroplasty: A Systematic Review. Reumatol Clin 2015; 11: 361-380.
15. Blom AW, Artz N, Beswick AD, Burston A, Dieppe P, Elvers KT, et al. Improving patients' experience and outcome of total joint replacement: the RESTORE programme. In: NIHR Journals Library Southampton (UK)2016.
16. Birch S, Stilling M, Mechlenburg I, Hansen TB. Effectiveness of a physiotherapist delivered cognitive-behavioral patient education for patients who undergoes operation for total knee arthroplasty: a protocol of a randomized controlled trial. BMC Musculoskelet Disord 2017; 18: 116.
17. Riddle DL, Keefe FJ, Ang D, J K, Dumenci L, Jensen MP, et al. A phase III randomized three-arm trial of physical therapist delivered pain coping skills training for patients with total knee arthroplasty: the KASTPain protocol. BMC Musculoskelet Disord 2012; 13: 149.
18. Judge A, Arden NK, Cooper C, Kassim Javaid M, Carr AJ, Field RE, et al. Predictors of outcomes of total knee replacement surgery. Rheumatology (Oxford, England) 2012; 51: 1804-1813.
19. Macrae WA. Chronic post-surgical pain: 10 years on. Br J Anaesth 2008; 101: 77-86.
20. Althaus A, Hinrichs-Rocker A, Chapman R, Arranz Becker O, Lefering R, Simanski C, et al. Development of a risk index for the prediction of chronic post-surgical pain. Eur J Pain 2012; 16: 901-910.
21. Wylde V, Lenguerrand E, Gooberman-Hill R, Beswick AD, Marques E, Noble S, et al. Effect of local anaesthetic infiltration on chronic postsurgical pain after total hip and knee replacement: the APEX randomised controlled trials. Pain 2015; 156: 1161-1170.
22. Chaparro LE, Smith SA, Moore RA, Wiffen PJ, Gilron I. Pharmacotherapy for the prevention of chronic pain after surgery in adults. Cochrane Database Syst Rev 2013: CD008307.

For peer review only - http://bmjopen.bmj.com/site/about/guidelines.xhtml

23. Artz N, Elvers KT, Lowe CM, Sackley C, Jepson P, Beswick AD. Effectiveness of physiotherapy exercise following total knee replacement: systematic review and meta-analysis. BMC Musculoskelet Disord 2015; 16: 15.

24. Fransen M, Nairn L, Bridgett L, Crosbie J, March L, Parker D, et al. Post-Acute Rehabilitation After Total Knee Replacement: A Multicenter Randomized Clinical Trial Comparing Long-Term Outcomes. Arthritis Care Res (Hoboken) 2017; 69: 192-200.

25. Moher D, Liberati A, Tetzlaff J, Altman DG, Group P. Preferred reporting items for systematic reviews and meta-analyses: the PRISMA statement. BMJ 2009; 339: b2535.

26. Marcano Belisario J, Tudor Car L, Reeves T, Gunn L, Car J. Search strategies to identify observational studies in MEDLINE and EMBASE (Protocol). Cochrane Database of Systematic Reviews 2013: MR000041.

27. Scottish Intercollegiate Guidelines Network. Search filters. http://www.sign.ac.uk/methodology/filters.html#obs Accesssed September 2016; Last updated on 23/01/17.

28. Higgins J, Green SE. Cochrane Handbook for Systematic Reviews of Interventions Version 5.1.0 [updated March 2011]. The Cochrane Collaboration, 2011. Available from www.cochrane-handbook.org. 2011.

29. Wylde V, Mackichan F, Bruce J, Gooberman-Hill R. Assessment of chronic post-surgical pain after knee replacement: Development of a core outcome set. European Journal of Pain 2015; 19: 611-620.

30. Slim K, Nini E, Forestier D, Kwiatkowski F, Panis Y, Chipponi J. Methodological index for non-randomized studies (minors): development and validation of a new instrument. ANZ J Surg 2003; 73: 712-716.

31. Wells G, Shea B, O'Connell D, Peterson J, Welch V, Losos M, et al. The Newcastle-Ottawa Scale (NOS) for assessing the quality if nonrandomized studies in meta-analyses. vol. 2017. Available from: URL: http://www.ohri.ca/programs/clinical_epidemiology/oxford.asp 2014.

32. Sterne JA, Hernan MA, Reeves BC, Savovic J, Berkman ND, Viswanathan M, et al. ROBINS-I: a tool for assessing risk of bias in non-randomised studies of interventions. BMJ 2016; 355: i4919.

33. Juni P, Witschi A, Bloch R, Egger M. The hazards of scoring the quality of clinical trials for meta-analysis. JAMA 1999; 282: 1054-1060.

For peer review only - http://bmjopen.bmj.com/site/about/guidelines.xhtml

34. Crosbie J, Naylor J, Harmer A, Russell T. Predictors of functional ambulation and patient perception following total knee replacement and short-term rehabilitation. Disability and rehabilitation 2010; 32: 1088-1098.

35. Edwards RR, Haythornthwaite JA, Smith MT, Klick B, Katz JN. Catastrophizing and depressive symptoms as prospective predictors of outcomes following total knee replacement. Pain research & management 2009; 14: 307-311.

36. Elson DW, Brenkel IJ. Predicting pain after total knee arthroplasty. The Journal of arthroplasty 2006; 21: 1047-1053.

37. Grosu I, Thienpont E, Dekock M, Scholtes JL, Lavand'homme P. Dynamic view of postoperative pain evolution after total knee arthroplasty: A prospective observational study. Minerva Anestesiologica 2016; 82: 274-283.

38. Nunez M, Nunez E, Luis del Val J, Ortega R, Segur JM, Hernandez MV, et al. Health-related quality of life in patients with osteoarthritis after total knee replacement: Factors influencing outcomes at 36 months of follow-up. Osteoarthritis and Cartilage 2007; 15: 1001-1007.

39. Nunez M, Lozano L, Nunez E, Segur JM, Sastre S, Macule F, et al. Total knee replacement and health-related quality of life: factors influencing long-term outcomes. Arthritis and rheumatism 2009; 61: 1062-1069.

40. Phillips JRA, Hopwood B, Arthur C, Stroud R, Toms AD. The natural history of pain and neuropathic pain after knee replacement: a prospective cohort study of the point prevalence of pain and neuropathic pain to a minimum three-year follow-up. The bone & joint journal 2014; 96-B: 1227-1233.

41. Pinto PR, McIntyre T, Ferrero R, Almeida A, Araujo-Soares V. Risk factors for moderate and severe persistent pain in patients undergoing total knee and hip arthroplasty: a prospective predictive study. PloS one 2013; 8: e73917.

42. Riis A, Rathleff MS, Jensen MB, Simonsen O. Low grading of the severity of knee osteoarthritis pre-operatively is associated with a lower functional level after total knee replacement: a prospective cohort study with 12 months' follow-up. The bone & joint journal 2014; 96-B: 1498-1502.

43. Sayers A, Wylde V, Lenguerrand E, Beswick AD, Gooberman-Hill R, Pyke M, et al. Rest Pain and Movement-Evoked Pain as Unique Constructs in Hip and Knee Replacements. Arthritis Care Res (Hoboken) 2016; 68: 237-245.

For peer review only - http://bmjopen.bmj.com/site/about/guidelines.xhtml

44. Stephens MAP, Druley JA, Zautra AJ. Older adults' recovery from surgery for osteoarthritis of the knee: Psychosocial resources and constraints as predictors of outcomes. Health Psychology 2002; 21: 377-383.

45. Thomazeau J, Rouquette A, Martinez V, Rabuel C, Prince N, Laplanche JL, et al. Predictive factors of chronic post-surgical pain at 6 months following knee replacement: Influence of postoperative pain trajectory and genetics. Pain Physician 2016; 19: E729-E741.

46. Kocic M, Stankovic A, Lazovic M, Dimitrijevic L, Stankovic I, Spalevic M, et al. Influence of fear of movement on total knee arthroplasty outcome. Annali Italiani Di Chirurgia 2015; 86: 148-155.

47. Veal FC, Bereznicki LRE, Thompson AJ, Peterson GM, Orlikowski C. Subacute Pain as a Predictor of Long-Term Pain Following Orthopedic Surgery An Australian Prospective 12 Month Observational Cohort Study. Medicine 2015; 94.

48. Bellamy N, Buchanan WW, Goldsmith CH, Campbell J, Stitt LW. Validation study of WOMAC: a health status instrument for measuring clinically important patient relevant outcomes to antirheumatic drug therapy in patients with osteoarthritis of the hip or knee. J Rheumatol 1988; 15: 1833-1840.

49. Insall JN, Dorr LD, Scott RD, Scott WN. Rationale of the Knee Society clinical rating system. Clinical Orthopaedics and Related Research 1989: 13-14.

50. Bafeta A, Dechartres A, Trinquart L, Yavchitz A, Boutron I, Ravaud P. Impact of single centre status on estimates of intervention effects in trials with continuous outcomes: meta-epidemiological study. BMJ 2012; 344: e813.

51. Murray DW, Britton AR, Bulstrode CJK. Loss to follow-up matters. J Bone Joint Surg Br 1997; 79-B: 254-257.

52. Kim J, Lonner JH, Nelson CL, Lotke PA. Response Bias: Effect on Outcomes Evaluation by Mail Surveys after Total Knee Arthroplasty. Journal of Bone and Joint Surgery - Series A 2004; 86: 15-21.

53. Stroup DF, Berlin JA, Morton SC, Olkin I, Williamson GD, Rennie D, et al. Meta-analysis of observational studies in epidemiology: a proposal for reporting. Meta-analysis Of Observational Studies in Epidemiology (MOOSE) group. JAMA 2000; 283: 2008-2012.

54. Dawson J, Fitzpatrick R, Murray D, Carr A. Questionnaire on the perceptions of patients about total knee replacement. The Journal of bone and joint surgery. British volume 1998; 80: 63-69.

For peer review only

For peer review only - http://bmjopen.bmj.com/site/about/guidelines.xhtml

55. Lopez-Olivo MA, Landon GC, Siff SJ, Edelstein D, Pak C, Kallen MA, et al. Psychosocial determinants of outcomes in knee replacement. Annals of the rheumatic diseases 2011; 70: 1775-1781.

56. Bellamy N. WOMAC: a 20-year experiential review of a patient-centered self-reported health status questionnaire. J Rheumatol 2002; 29: 2473-2476.

57. Harris K, Dawson J, Doll H, Field RE, Murray DW, Fitzpatrick R, et al. Can pain and function be distinguished in the Oxford Knee Score in a meaningful way? An exploratory and confirmatory factor analysis. Quality of Life Research: An International Journal of Quality of Life Aspects of Treatment, Care & Rehabilitation 2013; 22: 2561-2568.

58. van den Akker-Scheek I, Stevens M, Groothoff JW, Bulstra SK, Zijlstra W. Preoperative or postoperative self-efficacy: which is a better predictor of outcome after total hip or knee arthroplasty? Patient education and counseling 2007; 66: 92-99.

For peer review only

PRISMA 2009 Checklist

Section/topic	#	Checklist item	Reported on page #
**TITLE**			
Title	1	Identify the report as a systematic review, meta-analysis, or both.	1
**ABSTRACT**			
Structured summary	2	Provide a structured summary including, as applicable: background; objectives; data sources; study eligibility criteria, participants, and interventions; study appraisal and synthesis methods; results; limitations; conclusions and implications of key findings; systematic review registration number.	2
**INTRODUCTION**			
Rationale	3	Describe the rationale for the review in the context of what is already known.	3-4
Objectives	4	Provide an explicit statement of questions being addressed with reference to participants, interventions, comparisons, outcomes, and study design (PICOS).	3-4
**METHODS**			
Protocol and registration	5	Indicate if a review protocol exists, if and where it can be accessed (e.g., Web address), and, if available, provide registration information including registration number.	4
Eligibility criteria	6	Specify study characteristics (e.g., PICOS, length of follow-up) and report characteristics (e.g., years considered, language, publication status) used as criteria for eligibility, giving rationale.	4-5
Information sources	7	Describe all information sources (e.g., databases with dates of coverage, contact with study authors to identify additional studies) in the search and date last searched.	5
Search	8	Present full electronic search strategy for at least one database, including any limits used, such that it could be repeated.	Appendix 3
Study selection	9	State the process for selecting studies (i.e., screening, eligibility, included in systematic review, and, if applicable, included in the meta-analysis).	5-6
Data collection process	10	Describe method of data extraction from reports (e.g., piloted forms, independently, in duplicate) and any processes for obtaining and confirming data from investigators.	5-6
Data items	11	List and define all variables for which data were sought (e.g., PICOS, funding sources) and any assumptions and simplifications made.	5-6
Risk of bias in individual studies	12	Describe methods used for assessing risk of bias of individual studies (including specification of whether this was done at the study or outcome level), and how this information is to be used in any data synthesis.	6
Summary measures	13	State the principal summary measures (e.g., risk ratio, difference in means).	7
Synthesis of results	14	Describe the methods of handling data and combining results of studies, if done, including measures of consistency (e.g., I^2) for each meta-analysis.	7

For peer review only

For peer review only - http://bmjopen.bmj.com/site/about/guidelines.xhtml

PRISMA 2009 Checklist

Section/topic	#	Checklist item	Reported on page #
Risk of bias across studies	15	Specify any assessment of risk of bias that may affect the cumulative evidence (e.g., publication bias, selective reporting within studies).	6
Additional analyses	16	Describe methods of additional analyses (e.g., sensitivity or subgroup analyses, meta-regression), if done, indicating which were pre-specified.	7
**RESULTS**			
Study selection	17	Give numbers of studies screened, assessed for eligibility, and included in the review, with reasons for exclusions at each stage, ideally with a flow diagram.	7, Figure 1
Study characteristics	18	For each study, present characteristics for which data were extracted (e.g., study size, PICOS, follow-up period) and provide the citations.	7-8, Table 1
Risk of bias within studies	19	Present data on risk of bias of each study and, if available, any outcome level assessment (see item 12).	8, Table 2
Results of individual studies	20	For all outcomes considered (benefits or harms), present, for each study: (a) simple summary data for each intervention group (b) effect estimates and confidence intervals, ideally with a forest plot.	Tables 3-5
Synthesis of results	21	Present results of each meta-analysis done, including confidence intervals and measures of consistency.	N/A
Risk of bias across studies	22	Present results of any assessment of risk of bias across studies (see Item 15).	8, Table 2
Additional analysis	23	Give results of additional analyses, if done (e.g., sensitivity or subgroup analyses, meta-regression [see Item 16]).	N/A
**DISCUSSION**			
Summary of evidence	24	Summarize the main findings including the strength of evidence for each main outcome; consider their relevance to key groups (e.g., healthcare providers, users, and policy makers).	11-13
Limitations	25	Discuss limitations at study and outcome level (e.g., risk of bias), and at review-level (e.g., incomplete retrieval of identified research, reporting bias).	11-12
Conclusions	26	Provide a general interpretation of the results in the context of other evidence, and implications for future research.	12-13
**FUNDING**			
Funding	27	Describe sources of funding for the systematic review and other support (e.g., supply of data); role of funders for the systematic review.	14

From: Moher D, Liberati A, Tetzlaff J, Altman DG, The PRISMA Group (2009). Preferred Reporting Items for Systematic Reviews and Meta-Analyses: The PRISMA Statement. PLoS Med 6(7): e1000097. doi:10.1371/journal.pmed1000097

For peer review only - http://bmjopen.bmj.com/site/about/guidelines.xhtml

PRISMA 2009 Checklist

For more information, visit: www.prisma-statement.org.

For peer review only

Appendix 2: Search terms

MEDLINE (Ovid) (1946 to 17 October 2016)

1 Epidemiologic Studies/

2 exp Case-Control Studies/

3 exp Cohort Studies/

4 Cross-Sectional Studies/

5 (epidemiologic adj (study or studies)).ab,ti.

6 case control.ab,ti.

7 (cohort adj (study or studies)).ab,ti.

8 cross sectional.ab,ti.

9 cohort analy$.ab,ti.

10 (follow up adj (study or studies)).ab,ti.

11 longitudinal.ab,ti.

12 retrospective$.ab,ti.

13 prospective$.ab,ti.

14 (observ$ adj3 (study or studies)).ab,ti.

15 exp clinical study/

16 randomized controlled trial/

17 15 not 16

18 adverse effect?.ab,ti.

19 1 or 2 or 3 or 4 or 5 or 6 or 7 or 8 or 9 or 10 or 11 or 12 or 13 or 14 or 17 or 18

20 Arthroplasty, Replacement, Knee/

21 Knee Prosthesis/

22 (arthoplast$ adj3 knee$).mp. [mp=title, abstract, original title, name of substance word, subject heading word, keyword heading word, protocol supplementary concept word, rare disease supplementary concept word, unique identifier]

23 (knee$ adj3 replac$).mp. [mp=title, abstract, original title, name of substance word, subject heading word, keyword heading word, protocol supplementary concept word, rare disease supplementary concept word, unique identifier]

For peer review only - http://bmjopen.bmj.com/site/about/guidelines.xhtml

24 (knee adj3 implant$).mp. [mp=title, abstract, original title, name of substance word, subject heading word, keyword heading word, protocol supplementary concept word, rare disease supplementary concept word, unique identifier]

25 20 or 21 or 22 or 23 or 24

26 19 and 25

EMBASE (Ovid) (1980 to 17 October 2016)

1 Epidemiologic Studies/

2 exp Case-Control Studies/

3 exp Cohort Studies/

4 Cross-Sectional Studies/

5 (epidemiologic adj (study or studies)).ab,ti.

6 case control.ab,ti.

7 (cohort adj (study or studies)).ab,ti.

8 cross sectional.ab,ti.

9 cohort analy$.ab,ti.

10 (follow up adj (study or studies)).ab,ti.

11 longitudinal.ab,ti.

12 retrospective$.ab,ti.

13 prospective$.ab,ti.

14 (observ$ adj3 (study or studies)).ab,ti.

15 exp clinical study/

16 randomized controlled trial/

17 15 not 16

18 adverse effect?.ab,ti.

19 1 or 2 or 3 or 4 or 5 or 6 or 7 or 8 or 9 or 10 or 11 or 12 or 13 or 14 or 17 or 18

20 Arthroplasty, Replacement, Knee/

21 Knee Prosthesis/

22 (arthoplast$ adj3 knee$).mp. [mp=title, abstract, original title, name of substance word, subject heading word, keyword heading word, protocol supplementary concept word, rare disease supplementary concept word, unique identifier]

For peer review only - http://bmjopen.bmj.com/site/about/guidelines.xhtml
For peer review only

23 (knee$ adj3 replac$).mp. [mp=title, abstract, original title, name of substance word, subject heading word, keyword heading word, protocol supplementary concept word, rare disease supplementary concept word, unique identifier]

24 (knee adj3 implant$).mp. [mp=title, abstract, original title, name of substance word, subject heading word, keyword heading word, protocol supplementary concept word, rare disease supplementary concept word, unique identifier]

25 20 or 21 or 22 or 23 or 24

26 19 and 25

PsycINFO (inception [1806] to 23 March 2016

1. (knee$ adj3 arthoplast$).mp. [mp=title, abstract, heading word, table of contents, key concepts, original title, tests & measures]

2. (knee$ adj3 replac$).mp. [mp=title, abstract, heading word, table of contents, key concepts, original title, tests & measures]

3. (knee$ adj3 surg$).mp. [mp=title, abstract, heading word, table of contents, key concepts, original title, tests & measures]

4. (knee$ adj3 implant$).mp. [mp=title, abstract, heading word, table of contents, key concepts, original title, tests & measures]

5. (knee adj3 prosthe$).mp. [mp=title, abstract, heading word, table of contents, key concepts, original title, tests & measures]

6. 1 or 2 or 3 or 4 or 5

For peer review only

BMJ Open

Post-operative patient-related risk factors for chronic pain after total knee replacement: a systematic review

Journal:	*BMJ Open*
Manuscript ID	bmjopen-2017-018105.R1
Article Type:	Research
Date Submitted by the Author:	24-Aug-2017
Complete List of Authors:	Wylde, Vikki; University of Bristol, Musculoskeletal Research Unit<br>Beswick, Andrew; University of Bristol, School of Clinical Sciences<br>Dennis, Jane; University of Bristol, Musculoskeletal Research Unit<br>Gooberman-Hill, Rachael; University of Bristol, School of Clinical Sciences
**Primary Subject Heading**:	Surgery
Secondary Subject Heading:	Health services research
Keywords:	Knee < ORTHOPAEDIC & TRAUMA SURGERY, systematic review, Chronic post-surgical pain, Risk factors

SCHOLARONE™
Manuscripts

For peer review only

Post-operative patient-related risk factors for chronic pain after total knee replacement: a systematic review

Wylde V, Beswick AD, Dennis J, Gooberman-Hill R

Musculoskeletal Research Unit, School of Clinical Sciences, University of Bristol, UK

E-mail addresses

Vikki Wylde: v.wylde@bristol.ac.uk

Andrew Beswick: andy.beswick@bristol.ac.uk

Jane Dennis: jane.dennis@bristol.ac.uk

Rachael Gooberman-Hill: r.gooberman-hill@bristol.ac.uk

Corresponding author

Vikki Wylde

Musculoskeletal Research Unit, Learning and Research Building, Southmead Hospital, Bristol, BS10 5NB, UK. Telephone: 0117 414 7878; E-mail: V.Wylde@bristol.ac.uk

Running title: Risk factors for chronic pain after TKR

For peer review only - http://bmjopen.bmj.com/site/about/guidelines.xhtml

ABSTRACT

Objective

To identify post-operative patient-related risk factors for chronic pain after total knee replacement (TKR).

Design

The systematic review protocol was registered on PROSPERO (CRD42016041374). MEDLINE, Embase and PsycINFO were searched from inception to October 2016 with no language restrictions. Key articles were also tracked in ISI Web of Science. Cohort studies evaluating the association between patient-related factors in the first three months post-operative and pain at six months or longer after primary TKR surgery were included. Screening, data extraction and assessment of methodological quality were undertaken by two reviewers. The primary outcome was pain severity in the replaced knee measured with a patient-reported outcome measure at six months or longer after TKR. Secondary outcomes included adverse events and other aspects of pain recommended by the core outcome set for chronic pain after TKR.

Results

After removal of duplicates, 16,430 articles were screened, of which 805 were considered potentially relevant. After detailed evaluation of full-text articles, 14 studies with data from 1,168 participants were included. Post-operative patient-related factors included acute pain (eight studies), function (five studies) and psychosocial factors (four studies). The included studies had diverse methods for assessment of potential risk factors and outcomes and therefore narrative synthesis was conducted. For all post-operative factors, there was insufficient evidence to draw firm conclusions about the association with chronic pain after TKR. Selection bias was a potential risk for all studies, as none were reported to be conducted at multiple centres.

Conclusion

This systematic review found insufficient evidence to draw firm conclusions about the association between any post-operative patient-related factors and chronic pain after TKR. Further high-quality research is required to provide a robust evidence base on post-operative

For peer review only - http://bmjopen.bmj.com/site/about/guidelines.xhtml

risk factors, and inform the development and evaluation of targeted interventions to optimise patients' outcomes after TKR.

Key words: Total knee replacement, post-operative risk factors, chronic pain, systematic review

Strengths and limitations of this study

- This is the first systematic review of patient-related risk factors for chronic pain after total knee replacement.
- Meta-analysis was not possible due to heterogeneity in the assessment of risk factors and outcomes.
- We did not include studies that used a composite pain and function measure to assess outcome.

For peer review only

For peer review only - http://bmjopen.bmj.com/site/about/guidelines.xhtml

INTRODUCTION

Primary total knee replacement (TKR) is a common operation, with over 100,000 operations performed in the UK in 2015 [1, 2] and demand is projected to increase dramatically [3]. Patients choose to have a TKR to relieve chronic pain and improve functional ability [4] but approximately 20% of patients experience chronic post-surgical pain [5, 6] defined as pain present at three months after surgery [7]. The impact of chronic pain after TKR is considerable and patients may struggle to cope and adjust to this pain [8]. Provision of services for patients with chronic pain after TKR are patchy and inconsistent [9], with a lack of explicit access points [10]. A systematic review identified that only one intervention has been evaluated for the management of this condition: a single intraarticular botulinum toxin injection [11].

The identification of risk factors for chronic pain after TKR is a fundamental step in designing interventions to improve patient outcomes. Understanding the relevance of non-modifiable factors, such as sex and ethnicity, can help patients and clinicians work together to make informed decisions about TKR. Although some factors may not be modifiable, others may be amenable to intervention. Identification of modifiable patient-related risk factors is an important element in the development of interventions to improve outcomes after TKR. Previous systematic reviews have synthesised the literature on pre-operative risk factors for chronic pain after TKR [12-15]. These reviews have found evidence for a range of modifiable pre-operative patient-related risk factors, including pain intensity, catastrophising, mental health and co-morbidities. Pre-operative interventions have largely focused on exercise and education and have shown little long-term post-operative benefit [15]. Further interventions specifically targeting pain-related behavior, such as cognitive-behavioral patient education and pain coping skills training, are being evaluated [16, 17].

While the potential value for pre-operatively identifying at risk patients and targeting them with appropriate interventions is clear, multivariable models have been found to have low predictive power, explaining less than 10% of the variability in chronic pain [18]. An operation itself is an important risk factor for chronic pain [19], and factors relating to the operation and early recovery may be important risk factors. A risk index including pre-surgical variables and acute post-surgical pain had "fair" predictive power for the development of chronic post-surgical pain across diverse surgery types [20]. Therefore, in addition to evaluating pre-operative risk factors, it is important to consider post-surgical factors that may limit rehabilitation and recovery, and be associated with chronic pain. If

For peer review only - http://bmjopen.bmj.com/site/about/guidelines.xhtml

patients at risk of developing chronic pain could be identified in the early post-operative period, targeted interventions could be delivered, potentially as part of a comprehensive peri-operative care package, to prevent the development of chronic pain. Although trials evaluating the effectiveness of early post-operative interventions on reducing chronic pain have been conducted [21-24], no systematic review has yet evaluated post-operative risk factors for chronic pain after TKR. Therefore, the aim of this systematic review was to identify early post-operative patient-related risk factors for chronic pain after TKR.

METHODS

Protocol and registration

The protocol was registered on the international prospective register of systematic reviews (PROSPERO) on 6th July 2016 (reference: CRD42016041374). Conduct and reporting of this systematic review adheres to recommendations from PRISMA [25](Appendix 1).

Eligibility criteria

Studies were eligible for inclusion in the review if they met the following PICOS criteria:

Population: Adults undergoing primary TKR predominantly for osteoarthritis. Studies that included TKR patients combined with patients undergoing other orthopaedic procedures were included if separate results were available for TKR patients.

Exposure: Post-operative patient-related risk factors measured in the first three months after surgery. Patients with exposure were those with a risk factor (categorical variable) or higher level of risk factor (continuous variable). The focus of this review was on patient-related risk factors with the potential for modification or use in targeting care, and therefore studies which assessed clinical risk factors (e.g. length of stay, post-operative complications, or radiographic measurements) or analgesic use were excluded.

Comparator: Patients with absence of risk factor (categorical variable) or lower level of risk factor (continuous variable).

Outcome: Severity of pain in the replaced knee measured with a patient-reported outcome measure at six months or longer after TKR surgery.

Study design: Cohort studies that have explored the relationships between factors measured in the first three months post-operative and longer-term pain outcomes.

For peer review only - http://bmjopen.bmj.com/site/about/guidelines.xhtml

Information sources and searches

MEDLINE, Embase and PsycINFO were searched from inception to 17th October 2016. Searches were conducted by experienced systematic reviewers (AB and JD) based on established design filters [26, 27]. The search strategy combined terms relating to study design (e.g. cohort, epidemiological study) and population (e.g. knee replacement, knee arthroplasty). Full search strategies are provided in Appendix 2. No language restrictions were applied. Searches were supplemented with hand searching of reference lists and review articles, and key articles were tracked in ISI Web of Science. Conference abstracts were excluded. ClinicalTrials.gov was searched on the 18th August 2017 for ongoing observational studies and records screened in duplicate by two reviewers (JD and VW).

Study selection and data extraction

Bibliographic details of the articles identified were exported into EndNote X7 (Thomson Reuters) and duplicates removed. After an initial screening of titles and abstracts by one reviewer (AB) to remove clearly irrelevant studies, titles and abstracts were screened in duplicate by two reviewers (AB and VW). As recommended in the Cochrane Handbook [28], reviewers were 'over inclusive' at early stages and retained any potentially relevant studies. Full texts of all such reports were acquired and assessed for eligibility against the PICOS criteria in duplicate by two reviewers (AB and VW). Discrepancies were resolved in discussion with a third reviewer (JD). Data from articles that met the eligibility criteria were extracted into an Excel database by one reviewer (VW) with checking against source articles by a second reviewer (AB or JD). Extracted data comprised: country, date, setting, population, participant demographics, study methodology including statistical analysis, risk factors, time to follow-up, losses to follow-up, joint-specific pain outcomes, variables included in multivariable analyses and information relevant to assessment of study methodological quality.

Where necessary, authors of studies were contacted for further information to enable judgements about eligibility and/or to provide unpublished outcome data relevant to the review. If data from patients with TKR were combined with patients undergoing other orthopaedic procedures, separate data for patients with TKR were requested. If a combined pain and function outcome was reported, such as the Oxford Knee Score or WOMAC score,

For peer review only - http://bmjopen.bmj.com/site/about/guidelines.xhtml

separate pain-specific data were requested, e.g. the Oxford Knee Score pain subscale or WOMAC pain scale.

Outcomes

The primary outcome was pain severity in the replaced knee measured with a patient-reported outcome measure at six months or longer after TKR. Chronic post-surgical pain is defined as pain present at three months after surgery [7], however research has shown that most of the improvement in pain occurs in the first 3-6 months after TKR surgery [29-32]. Therefore, six months post-operative was deemed an appropriate time point to assess chronic pain. Secondary outcomes included adverse events and other aspects of pain recommended by the core outcome set for chronic pain after TKR [33]. These included pain interference with daily living, pain and physical functioning, temporal aspects of pain, pain description, emotional aspects of pain, use of pain medication, and satisfaction with pain relief. No limits were placed on the tools used to measure these outcomes.

Assessment of methodological quality of included studies

The Newcastle-Ottawa quality assessment scale [34] and ROBINS-I tool [35] are established tools for the assessment of risk of bias in randomised controlled trials and studies reporting non-randomised controlled comparisons. However, risk of bias assessment in systematic reviews of observational studies is less well established. The MINORs tool [36] has been developed, however this is a summative checklist, and as such risks rating reporting rather than conduct [37]. Therefore we developed a non-summative checklist for use in this review. This checklist consisted of four items to assess selection bias (inclusion of consecutive patients and representativeness), bias due to missing data (follow-up rates) and bias due to inadequate consideration of confounding (multivariable or univariable analysis). These items were informed by existing tools, including the MINORS, Newcastle-Ottawa quality assessment scale and the ROBINS-I tool. Each item was rated as adequate, not adequate or not reported. Each individual item rating is reported, rather than an overall score. Ratings of methodological quality for included studies were conducted independently by two reviewers (VW and JD) and any discrepancies were resolved in discussion with a third reviewer (AB).

Data synthesis

In the protocol, meta-analyses were planned if two or more studies assessed the same risk factor with suitable methodology. In comparing groups of patients with or without a risk

For peer review only - http://bmjopen.bmj.com/site/about/guidelines.xhtml

factor, outcomes adjusted for baseline patient factors would be considered in preference to unadjusted outcomes and the effect of non-adjustment would be explored in a sub-group analysis. If studies reported categorical pain outcomes, risk ratios would be used to summarise cohort studies and odds ratios for case-control studies. For risk factors reported as continuous variables, results of meta-analyses would be reported as mean differences or standardised mean differences, depending on the consistency of risk factor and outcome measures reported. We planned to explore the effect of non-adjustment for other variables in a sub-group analysis. Assessment of heterogeneity was planned using the chi-squared and I-squared statistic. The protocol stated that we would conduct a sensitivity analyses on methodological quality assessment.

At analysis stage, opportunities for meta-analysis were limited by heterogeneity in the assessment of risk factors and outcomes. Therefore, we undertook a descriptive narrative analysis, in keeping with the approach recommended by the Cochrane Handbook [28].

RESULTS

After removal of duplicates, 16,430 articles were screened, of which 857 were considered potentially relevant. After detailed evaluation of full-text articles, 14 studies with data from 1,613 participants were included [38-51](Figure 1). The most common reasons for excluding potentially relevant studies were because patient-related factors were not assessed and follow-up after TKR surgery was less than six months.

Study characteristics

An overview of study characteristics is provided in Table 1.

For peer review only

For peer review only - http://bmjopen.bmj.com/site/about/guidelines.xhtml

Table 1: Characteristics of included studies

Study	Dates of baseline data collection	Study design	Country	Participants recruited/at final follow-up	Mean/ median age	% female	Outcome measure	Duration of follow-up
Crosbie (2010)[38]	2005-2006	Cohort*	Australia	102/100	68	56%	WOMAC Pain	6 months
Edwards (2009) [39]	Not reported	Cohort	USA	43 in analysis	72	58%	VAS	12 months
Elson (2006) [40]	1995-1998	Case control	UK	622/402 knees	69	54%	AKSS pain question	5 years
Grosu (2016) [41]	2009-2010	Cohort	Belgium	114/68	66	66%	VDS	12 months
Nunez (2007) [42]	2000-2001	Cohort	Spain	88/67	75	81%	WOMAC Pain	3 years
Nunez (2009) [43]	2000	Cohort	Spain	142/112	67	77%	WOMAC Pain	7 years
Phillips (2014) [44]	2009-2010	Cohort	UK	96/80	71	56%	VAS	39-51 months
Pinto (2013) [45]	2009-2011	Cohort	Portugal	42 in analysis	66	77%	NRS	4-6 months
Riis (2014) [46]	2007-2009	Cohort	Denmark	176 /154	68	65%	AKSS pain question	12 months
Sayers (2016) [47]	2009-2012	Cohort*	UK	316/277	69	53%	WOMAC Pain	12 months
Stephens (2002) [48]	Not reported	Cohort	USA	71/63	67	54%	WOMAC Pain	6 months
Thomazeau (2016) [49]	2013	Cohort	France	109/104	69	72%	NRS	6 months
Kocic (2015) [50]	2007-2013	Cohort	Serbia	78/78	68	76%	NRS	6 months
Veal (2015) [51]	2013	Cohort	Australia	23 in analysis	Not available	Not available	VDS	12 months

*Retrospective analysis of RCT data

For peer review only - http://bmjopen.bmj.com/site/about/guidelines.xhtml

Of the 14 included studies, three were from the UK, two each from Australia, USA and Spain, and one study from Belgium, Denmark, France, Portugal and Serbia. Thirteen studies were conducted at a single centre and one study did not report the number of centres. Eleven of the studies were cohort studies, two were randomised controlled trials retrospectively analysed as cohort studies and one was a case-control study with prospective data collection. Sample sizes ranged from 23 to 402, with a median of 115 participants. One study included a small number of patients undergoing unicompartmental knee replacement but was included in the review as 83% of participants had TKR [49]. Follow-up assessments varied: four studies assessed outcomes at six months after TKR, five at 12 months and the remainder between 3-7 years post-operative. Pain at follow-up was evaluated using the WOMAC Pain scale [52](five studies), Numerical Rating Scale (NRS; three studies), Visual Analogue Scale (VAS; two studies), American Knee Society Score Pain question [53] (two studies), and Verbal Descriptor Scale (VDS; two studies). Secondary outcomes for the review relating to serious adverse events and other aspects of pain outcomes were infrequently reported and therefore not summarised.

Assessment of methodological quality of included studies

Ratings of methodological quality for the 14 included studies are provided in Table 2. Eight studies reported that consecutive patients were recruited, eight studies followed up >80% participants, and nine studies conducted multivariable analysis. All studies had issues relating to selection bias because none were reported as being conducted at multiple centres.

For peer review only

Table 2: Ratings of methodological quality for included studies

Study	Inclusion of consecutive patients	Representativeness (multi-centre adequate)	Percentage follow-up (>80% adequate)	Minimisation of potential confounding (multivariable analysis adequate)
Crosbie (2010)[38]	+	-	+	+
Edwards (2009) [39]		-	-	+
Elson (2006) [40]		-	-	-
Grosu (2016) [41]		-	-	-
Nunez (2007) [42]	+	-	-	+
Nunez (2009) [43]	+	-	-	+
Phillips (2014) [44]	+	-	+	-
Pinto[1] (2013) [45]	+	-	-	+
Riis (2014) [46]	+	-	+	+
Sayers (2016) [47]	+[2]	-	+	+
Stephens (2002) [48]			+	+
Thomazeau (2016) [49]	+	-	+	+
Kocic (2015) [50]		-	+	-
Veal[1] (2015) [51]		-	+	-

'+' adequate, '-' inadequate, 'blank' not reported

[1]For studies which authors provided data on patients with TKR, ratings are based on the study as reported in the article; [2] Information obtained through personal contact.

For peer review only

For peer review only - http://bmjopen.bmj.com/site/about/guidelines.xhtml

Patient-related post-operative risk factors

Patient-related post-operative risk factors were categorised into three groups: acute post-operative knee pain, knee function and psychosocial factors.

Acute post-operative knee pain

Eight studies including data from 737 participants evaluated the association between pain in the first three months after TKR and chronic pain (Table 3). Timing of acute postoperative pain was classified as: pain within the first postoperative week; pain between one and two weeks postoperative; and pain from two weeks to three months. Pain as a risk factor was assessed using the VAS (three studies), VDS (two studies), NRS (two studies), WOMAC Pain scale (one study) and PainDETECT (one study). Five studies conducted multivariable analysis, two studies conducted univariable analysis and for one study no statistical analysis was performed as data were provided by authors on a small subset of patients with TKR.

Pain severity on post-operative days 1-7

Four studies with data from 491 participants evaluated whether pain severity in the first week after surgery was associated with chronic pain [41, 45, 47, 49]. Two were at risk of bias due to missing data and one study was at risk of bias due to inadequate consideration of confounding. Methods used to assess pain included the VDS [41], VAS [47] and NRS [45, 49]. Three studies found that more severe acute post-operative pain was associated with more severe pain at 6-12 months after TKR [41, 47, 49], although in one study this association was attenuated completely after adjustment for pre-operative pain [47]. One study found no association between pain at 42 hours after surgery and the presence of chronic pain at 4-6 months [45].

Pain severity in post-operative days 8-14

Three studies with data from 191 participants evaluated whether pain severity on post-operative days 8-14 was associated with chronic pain [38, 41, 51]. One study was at risk of bias due to missing data and two studies were at risk of bias due to inadequate consideration of confounding. Pain was assessed in two studies with the VDS [41, 51] and in one with the WOMAC pain scale and VAS [38]. Pain on post-operative day eight and at two weeks was not found to be associated with chronic pain in two studies [38, 41], and descriptive data only were available for the study that evaluated pain on post-operative day 10 [51]. In the study

For peer review only - http://bmjopen.bmj.com/site/about/guidelines.xhtml

with low risk of bias apart from with regard to representativeness [38], pain severity at two weeks was not found to be associated with pain at six months after TKR

Pain severity between 2 weeks and 3 months post-operative

Five studies with data from 314 participants evaluated whether pain severity between two weeks and three months post-operative was associated with chronic pain after TKR [38, 39, 41, 44, 51]. Two studies were at risk of bias due to missing data and three studies were at risk of bias due to inadequate consideration of confounding. Methods to assess pain were the WOMAC pain scale [38], VAS [38, 39, 44] and VDS [41, 51]. In one study with risk of bias associated only with conduct at a single centre, pain severity at eight weeks post-operative was found to be associated with pain at six months post-operative when assessed with the WOMAC but not the VAS [38]. In one study with univariable analysis, pain severity assessed on day 30 was found to be associated with pain severity at six months but not 12 months after TKR [41]. The same study found that pain at three months post-operative was not associated with pain severity at six months and 12 months post-operative [41]. In another study, neuropathic pain at six weeks post-operative was found to be moderately associated with pain at 39-51 months after surgery [44]. In one study, there was no difference in pain at 12 months in patients with different average pain levels at six weeks [51]. However considering 'worst' pain, 7/14 patients with moderate to severe pain at six weeks reported moderate to severe pain at 12 months compared with 1/9 patients with none or mild pain at six weeks. A study which assessed global pain and night pain at one month and three months post-operative found that they were associated with global pain and night pain respectively at a future time point (six months and 12 months) [39].

For peer review only - http://bmjopen.bmj.com/site/about/guidelines.xhtml

Table 3: Studies evaluating acute post-operative knee pain as a risk factor for chronic pain after TKR

Author and date	Number in analysis	Risk factor measurement	Outcome(s)	Univariable or multivariable analysis	Association	Results summary
Edwards 2009 [39]	43	Global pain VAS at 1 month and 3 months<br><br>Night pain VAS at 1 month and 3 months	Global pain VAS at 6 and 12 months<br><br>Night pain VAS at 6 and 12 months	Multivariable generalised estimating equation model	Yes<br><br>Yes	Global pain at a previous time point was a predictor of global pain at a future time point (estimate=0.43, SE=0.08, t=5.8, p<0.001)<br><br>Night pain at a previous time point was a predictor of night pain at a future time point (estimate=0.32, SE=0.08, t=3.8, p<0.001)
Crosbie 2010 [38]	100	WOMAC Pain Scale at 2 weeks<br><br>VAS at 2 weeks<br><br>WOMAC Pain at 8 weeks<br><br>VAS at 8 weeks	WOMAC Pain scale at 6 months	Multivariable linear regression	No<br><br>No<br><br>Yes<br><br>No	Not significant, results not reported<br><br>Not significant, results not reported<br><br>Beta coefficient = +0.25 ± 0.07<br><br>Not significant, results not reported
Pinto 2013 [45]	42	NRS at 48 hours	NRS at 4-6 months	Hierarchical logistic regression	No	Exp(B) =0.998 (95% CI 0.623-1.601), p value=0.995
Phillips 2014 [44]	80	PainDETECT at 6 weeks	Pain VAS at 39-51 months	Univariable correlation	Yes	PainDETECT at 6 weeks correlated moderately with VAS pain scores (r=0.53)
Veal 2015 [51]	23	VDS for average pain at 10 days	VDS for average pain at 12 months	N/A – statistical analysis inappropriate as data provided by authors on a small subset of patients	N/A	11 patients had none/mild pain at 10 days, none of these patients had severe/moderate pain at 12 months.<br><br>12 patients had moderate/severe pain at 10 days, 2 of these patients had moderate/severe at 12 months.

For peer review only - http://bmjopen.bmj.com/site/about/guidelines.xhtml

		VDS for worst pain at 10 days	VDS for worse pain at 12 months			2 patients had none/mild pain at 10 days, none of these patients had severe/moderate pain at 12 months.<br><br>21 patients had moderate/severe pain at 10 days, 8 of these patients had moderate/severe at 12 months.
		VDS for average pain at 6 week	VDS for average pain at 12 months			17 patients had none/mild pain at 6 weeks, 1 of these patients had moderate/severe pain at 12 months.<br><br>6 patients had moderate/severe pain at 6 weeks, 1 of these patients had moderate/severe at 12 months.
		VDS for worst pain at 6 weeks	VDS for worse pain at 12 months			9 patients had none/mild pain at 6 weeks, 1 of these patients had severe/moderate pain at 12 months.<br><br>14 patients had moderate/severe pain at 6 weeks, 7 of these patients had moderate/severe at 12 months.
Grosu 2016 [41]	68	VDS on days 1,2 and 3 (cumulative value of maximal pain intensity)	VDS at 6 months	Univariable correlation	Yes	r=0.350; p value = 0.009
			VDS at 12 months		Yes	r=0.350; p value = 0.009
		VDS on day 8	VDS at 6 months		No	Not significant, results not reported
			VDS at 12 months		No	Not significant, results not reported
		VDS on day 30	VDS at 6 months		Yes	r=0.310, p=0.013

For peer review only - http://bmjopen.bmj.com/site/about/guidelines.xhtml

			VDS at 12 months		No	Not significant, results not reported
		VDS at 3 months	VDS at 6 months		No	Not significant, results not reported
			VDS at 12 months		No	Not significant, results not reported
Sayers 2016 [47]	277	VAS for pain on rest on days 1,2 and 3 (combined)	WOMAC Pain at 12 months	Multivariable structural equation modelling	Yes	Beta=0.222, SE=0.058, 95% CI = 0.109, 0.336, p value = 0.0001<br><br>When pre-operative pain added: Beta=0.09, 95% CI = -0.09, 0.27, p value = 0.332
		VAS for pain on movement on days 1,2 and 3(combined)			Yes	Beta=0.140, SE=0.044, 95% CI = 0.054, 0.226, p value = 0.0014<br><br>When pre-operative pain added: Beta=0.00, 95% CI = -0.14, 0.15, p value = 0.955
Thomazeau 2016 [49]	104	NRS on days 1-4	NRS at 6 months	Multivariate logistic regression	Yes	Patients with high intensity acute post-operative pain (defined though Latent Class Growth analysis) were more likely to have pain at 6 months than patients with low intensity acute post-operative pain (OR=4.23, 95% CI=1.39-12.88, p-value=0.011)

For peer review only - http://bmjopen.bmj.com/site/about/guidelines.xhtml

Knee function

Five studies including data from 835 participants evaluated the association between post-operative knee function and chronic pain after TKR (Table 4). Three studies were at risk of bias due to missing data and one study was at risk of bias due to inadequate consideration of confounding. Assessment of knee function varied and included range of motion, ambulatory status, WOMAC Function, six minute walk test and stair ascent speed.

Four studies including data from 735 participants evaluated whether function at hospital discharge was associated with chronic pain after TKR [40, 42, 43, 46]. Two of these studies assessed range of motion [40, 46] and two assessed ambulatory status at discharge [42, 43]; none found an association. One study, at low risk of bias except inclusion of a single centre, with 100 patients evaluated whether function at two weeks and eight weeks, assessed using three different methods, was associated with WOMAC Pain scores at six months post-operative [38]. This study found that WOMAC Function score at two weeks, but not eight weeks, was associated with chronic pain; six minute walk test at both two weeks and eight weeks was associated with chronic pain; stair ascent speed at two and eight weeks was not associated with chronic pain.

For peer review only

For peer review only - http://bmjopen.bmj.com/site/about/guidelines.xhtml

Table 4: Studies evaluating post-operative knee function as a risk factor for chronic pain after TKR

Author and date	Number in analysis	Risk factor measurement	Outcome	Univariable or multivariable analysis	Association	Results summary
Elson and Brenkel 2006 [40]	402 knees	Range of motion (active and passive) at hospital discharge	AKSS Pain at 5 years	Univariable analysis	No	Not significant, results not reported
Nunez 2007 [42]	67	Ambulatory status at hospital discharge	WOMAC Pain at 3 years	Multivariable linear regression	No	Not significant, results not reported
Nunez 2009 [43]	112	Ambulatory status at hospital discharge	WOMAC Pain at 7 years	Multivariable linear regression	No	Not significant, results not reported
Crosbie 2010 [38]	100	WOMAC Function at 2 weeks<br><br>6 minute walk test at 2 weeks<br><br>Stair ascent speed at 2 weeks<br><br>WOMAC Function at 8 weeks<br><br>6 minute walk test at 8 weeks<br><br>Stair ascent speed at 8 weeks	WOMAC Pain at 6 months	Multivariable linear regression	Yes<br><br>Yes<br><br>No<br><br>No<br><br>Yes<br><br>No	Beta coefficient = +0.06, SE = ± 0.02.<br><br>Beta coefficient = -0.05, SE = ± 0.01.<br><br>Not significant, results not reported<br><br>Not significant, results not reported<br><br>Beta coefficient = -0.04, SE = ± 0.01.<br><br>Not significant, results not reported
Riis 2014 [46]	154	Range of flexion (active) at hospital discharge	AKSS Pain at 12 months	Multivariable binary logistic regression	No	OR 1.00 (95% CI 0.99 to 1.04), p=0.698

For peer review only - http://bmjopen.bmj.com/site/about/guidelines.xhtml

Psychosocial factors

Four studies including data from 226 participants evaluated the association between post-operative psychological factors and chronic pain after TKR (Table 5). Two studies were at risk of bias due to missing data and one study was at risk of bias due to inadequate consideration of confounding. Risk factors assessed included catastrophising, depression, social support, coping skills, fear of movement and anxiety. In one study, catastrophising at a previous time point was a risk factor for night pain, but not global pain, at a future time point [39]. In the same study, depression was found to be a risk factor for global pain but not night pain. Another study assessing risk factors at six weeks post-operative found that perceived positive social support was associated with less chronic pain, negative social support with more chronic pain, and no association between coping and pain at six months after TKR [48]. Patients with a high fear of movement at two weeks post-operative reported more pain at six months than those with a low fear of movement [50]. Greater anxiety at 48 hours after surgery was found to be associated with a higher risk of having a pain score of >3 on a NRS at 4-6 months after TKR [45].

Ongoing studies

Searches of ClinicalTrials.gov identified five ongoing studies which are collecting data on patient-related post-operative risk factors and pain outcomes at six months or longer after TKR. An overview of these studies is provided in Appendix 3.

For peer review only

Table 5: Studies evaluating post-operative psychological factors as risk factors for chronic pain after TKR

Author and date	Number in analysis	Risk factor measurement	Outcome(s)	Univariable or multivariable analysis	Association	Results summary
Stephens 2002 [48]	63	Perceived positive social support (MOS Social Support survey) at 6 weeks	WOMAC Pain at 6 months	Multivariable hierarchical multiple regression	Yes	Beta=-0.29, SE=0.09, p≤0.05
		Perceived negative social support (4 items) at 6 weeks			Yes	Beta=-0.27, SE=0.14, p≤0.05
		Active coping (Vanderbilt Multidimensional Pain Coping Inventory Active Coping scale) at 6 weeks			No	Beta=-0.14, SE=0.01
		Avoidant coping (Vanderbilt Multidimensional Pain Coping Inventory Avoidant Coping scale) at 6 weeks			No	Beta=0.21, SE=0.01
Edwards 2009 [39]	43	Catastrophising (Coping Strategies Questionnaire catastrophizing subscale) at 1 month and 3 months	Global pain VAS at 6 and 12 months	Multivariable generalised estimating equation model	No	Catastrophising at a previous time point was not a predictor of global pain at a future time point (estimate=2.1, SE=2.2, t=0.9, p=0.35)
			Night pain VAS at 6 and 12 months		Yes	Catastrophising at a previous time was a predictor of nighttime pain at a future time point (estimate=5.1, SE=2.5, t=2.0, p=0.04).
		Depression (Centre for Epidemiological Studies Depression Scale at 1 month and 3 months)	Global pain VAS at 6 and 12 months		Yes	Depression at a previous time point was a predictor of global pain at a future time point (estimate=0.67, SE=0.30, t=2.2, p=0.03)

For peer review only - http://bmjopen.bmj.com/site/about/guidelines.xhtml

			Night pain VAS at 6 and 12 months		No	Depression at a previous time point was not a predictor of nighttime pain at a future time point (estimate=0.40, SE=0.33, t=1.2, p=0.24).
Pinto 2013 [45]	42	Anxiety scale (Hospital Anxiety and Depression Scale) at 48 hours	NRS at 4-6 months	Hierarchical logistic regression	Yes	Exp(B) = 1.713 (95% CI 1.104-2.657), p value=0.016
Kocic 2015 [50]	78	Fear of movement (Tampa Scale of Kinesiophobia) at 2 weeks	NRS at 6 months	Univariable comparison of means	Yes	Patients with high fear of movement had more pain (mean=3.24, SD=1.98) than patients with low fear of movement (mean=1.81, SD=1.5), p=0.0035

For peer review only - http://bmjopen.bmj.com/site/about/guidelines.xhtml

DISCUSSION

This is the first systematic review to evaluate post-operative patient-related risk factors for chronic pain after TKR. Fourteen cohort studies were identified which evaluated the association between patient-related factors measured in the first three months post-operative and pain severity measured with a patient-reported outcome measure at six months or longer after primary TKR. Post-operative factors assessed included pain (eight studies), function (five studies) and psychosocial factors (four studies).

For all post-operative patient-related factors, there was insufficient evidence to draw firm conclusions on the association with chronic pain after TKR. When reviewing observational cohort studies, it is essential to consider issues that may introduce bias and lead to potentially misleading results and their interpretation. The key issues relate to generalisability, incomplete follow-up and accounting for confounding factors. Regarding generalisability, findings from single-centre and multi-centre studies can differ [54], and one potential factor contributing to this difference is the recruitment of a more homogeneous population in single-centre studies. The population may be highly selected and therefore have limited validity external to the study setting. Losses to follow-up represent another cause of bias as patients who do not complete longer-term assessments may have poorer outcomes [55, 56]. In this review, six studies had data on <80% participants at follow-up. The methodological quality of five studies was limited by the lack of multivariable analysis to minimise the impact of potential confounding on results. In studies with no risk of bias other than patient selection, there was a suggestion that chronic pain was associated with increased acute post-operative pain during the hospital stay [47, 49]. However, in one of these studies, a comprehensive assessment of pain relationships over time suggested that the association was largely explained by pre-operative pain [47]. For later pain assessments, one study did not identify consistent associations between post-operative pain and chronic pain [38].

This review has strengths and weaknesses which should be considered when interpreting the results. While our search terms were broad to identify cohort studies which involved patients with TKR, three studies were identified through methods other than the main searches. This is a recognised issue in the identification of observational studies [57] and highlights the importance in bibliographic databases of appropriate indexing and use of keywords. It is possible that studies including general orthopaedic or surgical populations may have included patients with TKR, and these may not have been identified. However, when these studies were identified, we contacted authors and data for patients with TKR were provided for two

For peer review only - http://bmjopen.bmj.com/site/about/guidelines.xhtml

studies [45, 51]. The primary outcome of interest in this review was pain at six months or longer after TKR, and therefore we did not include studies that used a composite pain and function measure to assess outcome, for example the total Oxford Knee Score [58] or WOMAC [52]. This is because when such composite measures are reported without any separation of pain from function it is not possible to use the scores to assess pain *per se*. Pre-operative risk factors for post-operative pain and functional limitations are different [18, 59], and therefore it is important to assess pain and function as distinct outcomes. Separate pain and function scores can be calculated for the most commonly used patient-reported outcome measures, the WOMAC [60] and the Oxford Knee Score [61], and future studies would benefit from analysing these outcomes separately. Research on post-operative risk factors is limited by heterogeneity in how and when risk factors and outcomes are assessed. If greater standardisation could be achieved, such as through the implementation of core outcome sets [33], future systematic reviews may be able to pool data in meta-analysis to provide evidence for post-operative patient-related risk factors for chronic pain after TKR.

Much of the research evaluating risk factors for outcomes after TKR has focused on the pre-operative period rather than the period after surgery [12]. Numerous pre-operative patient-related factors and their association to chronic pain have been evaluated, including knee pain severity and duration, pain at other sites, comorbidities, function, depression, social support, anxiety, fear of movement, pessimism and quality of life [12]. In comparison, our review found that the current extent of research into post-operative risk factors is narrow, and further research is needed. Searches of ClinicalTrials.gov found that a number of studies are ongoing in this field, suggesting the evidence-base will continue to grow and develop. Assessing potential post-operative risk factors is important as some factors may be more associated with outcome when measured in the post-operative period, rather than the pre-operative period [62]. Prediction of chronic post-surgical pain has been found to be strongest when assessing both pre-operative and post-operative risk factors [20]. Factors specific to the post-operative recovery period, such as acute post-operative pain, and factors which span the peri-operative period, such as anxiety, have the potential to influence outcomes. Identification of both pre-operative and post-operative risk factors could inform the development of comprehensive care packages to improve outcomes.

Despite the lack of sufficient evidence about post-operative risk factors, research has evaluated whether early post-operative interventions improve longer-term outcomes after TKR. The long-term effects of pharmacological interventions to reduce pain severity in the

For peer review only - http://bmjopen.bmj.com/site/about/guidelines.xhtml

For peer review only

early post-operative period have been evaluated, both in patients undergoing TKR and other surgical procedures [21, 22]. While effective at reducing acute post-operative pain, numerous peri-operative pharmacotherapies are not effective at preventing chronic post-surgical pain. Similarly, outpatient physiotherapy interventions to improve early post-operative function have little effect on long-term pain [23, 24]. This may be because acute post-operative pain and functional limitations are not risk factors for chronic pain after TKR or it may be that these interventions require evaluation in trials that are focused on high-risk patients. However, before evaluation of such stratified models of care is possible, more research is needed to identify post-operative patient-related risk factors for chronic pain after TKR.

In conclusion, this systematic review found insufficient evidence to draw conclusions about the association between any post-operative patient-related factor and chronic pain after TKR. To complement this research, systematic reviews are ongoing to evaluate the effectiveness of pre-operative, peri-operative and post-operative interventions in preventing chronic pain after TKR (PROSPERO reference CRD42017041382). Further high-quality research is required to provide robust evidence on post-operative risk factors, and inform the development and evaluation of targeted interventions to optimise patients' outcomes after TKR.

For peer review only

For peer review only - http://bmjopen.bmj.com/site/about/guidelines.xhtml

Acknowledgements

We would like to thank all the study authors who took the time to reply to our requests for further clarification or additional data.

Author contributions

All authors contributed to the concept and design of the study. ADB, JD and VW contributed to the acquisition and analysis of data. VW drafted the article and ADB, JD and RGH revised it critically for important intellectual content. VW and ADB take responsibility for the integrity of the work as a whole, from inception to finished article.

Funding

This article presents independent research funded by the National Institute for Health Research (NIHR) under its Programme Grants for Applied Research programme (RP-PG-0613-20001). The views expressed are those of the authors and not necessarily those of the NHS, the NIHR or the Department of Health. The funder had no role in the study design, collection, analysis and interpretation of data; in the writing of the manuscript; or in the decision to submit the manuscript for publication.

Competing interests

The authors have no conflicts of interest to declare.

Data sharing statement

No additional data are available.

Figure legends

Figure 1: Systematic review flow diagram

For peer review only

References

1. National Joint Registry for England W, Northern Ireland and the Isle of Man. 13th Annual Report Hemel Hempstead: NJR Centre.2016.
2. Scottish Arthroplasty Project. Biennial report NHS National Services Scotland2016.
3. Maradit Kremers H, Larson DR, Crowson CS, Kremers WK, Washington RE, Steiner CA, et al. Prevalence of Total Hip and Knee Replacement in the United States. J Bone Joint Surg Am 2015; 97: 1386-1397.
4. Scott CE, Bugler KE, Clement ND, MacDonald D, Howie CR, Biant LC. Patient expectations of arthroplasty of the hip and knee. J Bone Joint Surg Br 2012; 94: 974-981.
5. Beswick AD, Wylde V, Gooberman-Hill R, Blom A, Dieppe P. What proportion of patients report long-term pain after total hip or knee replacement for osteoarthritis? A systematic review of prospective studies in unselected patients. BMJ Open 2012; 2: e000435.
6. Wylde V, Hewlett S, Learmonth ID, Dieppe P. Persistent pain after joint replacement: Prevalence, sensory qualities, and postoperative determinants. Pain 2011; 152: 566-572.
7. Werner MU, Kongsgaard UE. I. Defining persistent post-surgical pain: is an update required? Br J Anaesth 2014; 113: 1-4.
8. Jeffery AE, Wylde V, Blom AW, Horwood JP. "It's there and I'm stuck with it": patients' experiences of chronic pain following total knee replacement surgery. Arthritis Care Res (Hoboken) 2011; 63: 286-292.
9. Wylde V, Mackichan, Dixon S, Gooberman-Hill R. Service provision for patients with chronic post-surgical pain after total knee replacement: An evaluation of current practice. Journal of Pain Management 2014; 7: 147-154.
10. MacKichan F, Wylde V, Gooberman-Hill R. Pathways Through Care for Long-Term Pain After Knee Replacement: A Qualitative Study With Healthcare Professionals. Musculoskeletal Care 2015.
11. Beswick AD, Wylde V, Gooberman-Hill R. Interventions for the prediction and management of chronic postsurgical pain after total knee replacement: systematic review of randomised controlled trials. BMJ Open 2015; 5: e007387.

For peer review only - http://bmjopen.bmj.com/site/about/guidelines.xhtml

12. Lewis GN, Rice DA, McNair PJ, Kluger M. Predictors of persistent pain after total knee arthroplasty: a systematic review and meta-analysis. British journal of anaesthesia 2015; 114: 551-561.
13. Baert IA, Lluch E, Mulder T, Nijs J, Noten S, Meeus M. Does pre-surgical central modulation of pain influence outcome after total knee replacement? A systematic review. Osteoarthritis Cartilage 2016; 24: 213-223.
14. Hernandez C, Diaz-Heredia J, Berraquero ML, Crespo P, Loza E, Ruiz Iban MA. Pre-operative Predictive Factors of Post-operative Pain in Patients With Hip or Knee Arthroplasty: A Systematic Review. Reumatol Clin 2015; 11: 361-380.
15. Blom AW, Artz N, Beswick AD, Burston A, Dieppe P, Elvers KT, et al. Improving patients' experience and outcome of total joint replacement: the RESTORE programme. In: NIHR Journals Library Southampton (UK)2016.
16. Birch S, Stilling M, Mechlenburg I, Hansen TB. Effectiveness of a physiotherapist delivered cognitive-behavioral patient education for patients who undergoes operation for total knee arthroplasty: a protocol of a randomized controlled trial. BMC Musculoskelet Disord 2017; 18: 116.
17. Riddle DL, Keefe FJ, Ang D, J K, Dumenci L, Jensen MP, et al. A phase III randomized three-arm trial of physical therapist delivered pain coping skills training for patients with total knee arthroplasty: the KASTPain protocol. BMC Musculoskelet Disord 2012; 13: 149.
18. Judge A, Arden NK, Cooper C, Kassim Javaid M, Carr AJ, Field RE, et al. Predictors of outcomes of total knee replacement surgery. Rheumatology (Oxford, England) 2012; 51: 1804-1813.
19. Macrae WA. Chronic post-surgical pain: 10 years on. Br J Anaesth 2008; 101: 77-86.
20. Althaus A, Hinrichs-Rocker A, Chapman R, Arranz Becker O, Lefering R, Simanski C, et al. Development of a risk index for the prediction of chronic post-surgical pain. Eur J Pain 2012; 16: 901-910.
21. Wylde V, Lenguerrand E, Gooberman-Hill R, Beswick AD, Marques E, Noble S, et al. Effect of local anaesthetic infiltration on chronic postsurgical pain after total hip and knee replacement: the APEX randomised controlled trials. Pain 2015; 156: 1161-1170.
22. Chaparro LE, Smith SA, Moore RA, Wiffen PJ, Gilron I. Pharmacotherapy for the prevention of chronic pain after surgery in adults. Cochrane Database Syst Rev 2013: CD008307.

For peer review only

For peer review only - http://bmjopen.bmj.com/site/about/guidelines.xhtml

23. Artz N, Elvers KT, Lowe CM, Sackley C, Jepson P, Beswick AD. Effectiveness of physiotherapy exercise following total knee replacement: systematic review and meta-analysis. BMC Musculoskelet Disord 2015; 16: 15.

24. Fransen M, Nairn L, Bridgett L, Crosbie J, March L, Parker D, et al. Post-Acute Rehabilitation After Total Knee Replacement: A Multicenter Randomized Clinical Trial Comparing Long-Term Outcomes. Arthritis Care Res (Hoboken) 2017; 69: 192-200.

25. Moher D, Liberati A, Tetzlaff J, Altman DG, Group P. Preferred reporting items for systematic reviews and meta-analyses: the PRISMA statement. BMJ 2009; 339: b2535.

26. Marcano Belisario J, Tudor Car L, Reeves T, Gunn L, Car J. Search strategies to identify observational studies in MEDLINE and EMBASE (Protocol). Cochrane Database of Systematic Reviews 2013: MR000041.

27. Scottish Intercollegiate Guidelines Network. Search filters. http://www.sign.ac.uk/methodology/filters.html#obs Accesssed September 2016; Last updated on 23/01/17.

28. Higgins J, Green SE. Cochrane Handbook for Systematic Reviews of Interventions Version 5.1.0 [updated March 2011]. The Cochrane Collaboration, 2011. Available from www.cochrane-handbook.org. 2011.

29. Lenguerrand E, Wylde V, Gooberman-Hill R, Sayers A, Brunton L, Beswick AD, et al. Trajectories of Pain and Function after Primary Hip and Knee Arthroplasty: The ADAPT Cohort Study. PLoS One 2016; 11: e0149306.

30. Davis AM, Perruccio AV, Ibrahim S, Hogg-Johnson S, Wong R, Streiner DL, et al. The trajectory of recovery and the inter-relationships of symptoms, activity and participation in the first year following total hip and knee replacement. Osteoarthritis Cartilage 2011; 19: 1413-1421.

31. Halket A, Stratford PW, Kennedy DM, Woodhouse LJ. Using hierarchical linear modeling to explore predictors of pain after total hip and knee arthroplasty as a consequence of osteoarthritis. J Arthroplasty 2010; 25: 254-262.

32. Wylde V, Dixon S, Miller L, Whitehouse MR, Blom AW. 5 Year Outcomes and Survivorship of the Triathlon Total Knee Replacement: A Cohort Study. Acta Orthop Belg in press.

For peer review only - http://bmjopen.bmj.com/site/about/guidelines.xhtml

33. Wylde V, Mackichan F, Bruce J, Gooberman-Hill R. Assessment of chronic post-surgical pain after knee replacement: Development of a core outcome set. European Journal of Pain 2015; 19: 611-620.

34. Wells G, Shea B, O'Connell D, Peterson J, Welch V, Losos M, et al. The Newcastle-Ottawa Scale (NOS) for assessing the quality if nonrandomized studies in meta-analyses. vol. 2017. Available from: URL: http://www.ohri.ca/programs/clinical_epidemiology/oxford.asp 2014.

35. Sterne JA, Hernan MA, Reeves BC, Savovic J, Berkman ND, Viswanathan M, et al. ROBINS-I: a tool for assessing risk of bias in non-randomised studies of interventions. BMJ 2016; 355: i4919.

36. Slim K, Nini E, Forestier D, Kwiatkowski F, Panis Y, Chipponi J. Methodological index for non-randomized studies (minors): development and validation of a new instrument. ANZ J Surg 2003; 73: 712-716.

37. Juni P, Witschi A, Bloch R, Egger M. The hazards of scoring the quality of clinical trials for meta-analysis. JAMA 1999; 282: 1054-1060.

38. Crosbie J, Naylor J, Harmer A, Russell T. Predictors of functional ambulation and patient perception following total knee replacement and short-term rehabilitation. Disability and rehabilitation 2010; 32: 1088-1098.

39. Edwards RR, Haythornthwaite JA, Smith MT, Klick B, Katz JN. Catastrophizing and depressive symptoms as prospective predictors of outcomes following total knee replacement. Pain Research & Management 2009; 14: 307-311.

40. Elson DW, Brenkel IJ. Predicting Pain After Total Knee Arthroplasty. The Journal of Arthroplasty 2006; 21: 1047-1053.

41. Grosu I, Thienpont E, Dekock M, Scholtes JL, Lavand'homme P. Dynamic view of postoperative pain evolution after total knee arthroplasty: A prospective observational study. Minerva Anestesiologica 2016; 82: 274-283.

42. Nunez M, Nunez E, Luis del Val J, Ortega R, Segur JM, Hernandez MV, et al. Health-related quality of life in patients with osteoarthritis after total knee replacement: Factors influencing outcomes at 36 months of follow-up. Osteoarthritis and Cartilage 2007; 15: 1001-1007.

43. Nunez M, Lozano L, Nunez E, Segur JM, Sastre S, Macule F, et al. Total knee replacement and health-related quality of life: factors influencing long-term outcomes. Arthritis and rheumatism 2009; 61: 1062-1069.

For peer review only - http://bmjopen.bmj.com/site/about/guidelines.xhtml

44. Phillips JRA, Hopwood B, Arthur C, Stroud R, Toms AD. The natural history of pain and neuropathic pain after knee replacement: a prospective cohort study of the point prevalence of pain and neuropathic pain to a minimum three-year follow-up. The bone & joint journal 2014; 96-B: 1227-1233.

45. Pinto PR, McIntyre T, Ferrero R, Almeida A, Araujo-Soares V. Risk factors for moderate and severe persistent pain in patients undergoing total knee and hip arthroplasty: a prospective predictive study. PloS one 2013; 8: e73917.

46. Riis A, Rathleff MS, Jensen MB, Simonsen O. Low grading of the severity of knee osteoarthritis pre-operatively is associated with a lower functional level after total knee replacement: a prospective cohort study with 12 months' follow-up. The bone & joint journal 2014; 96-B: 1498-1502.

47. Sayers A, Wylde V, Lenguerrand E, Beswick AD, Gooberman-Hill R, Pyke M, et al. Rest Pain and Movement-Evoked Pain as Unique Constructs in Hip and Knee Replacements. Arthritis Care Res (Hoboken) 2016; 68: 237-245.

48. Stephens MAP, Druley JA, Zautra AJ. Older adults' recovery from surgery for osteoarthritis of the knee: Psychosocial resources and constraints as predictors of outcomes. Health Psychology 2002; 21: 377-383.

49. Thomazeau J, Rouquette A, Martinez V, Rabuel C, Prince N, Laplanche JL, et al. Predictive factors of chronic post-surgical pain at 6 months following knee replacement: Influence of postoperative pain trajectory and genetics. Pain Physician 2016; 19: E729-E741.

50. Kocic M, Stankovic A, Lazovic M, Dimitrijevic L, Stankovic I, Spalevic M, et al. Influence of fear of movement on total knee arthroplasty outcome. Annali Italiani Di Chirurgia 2015; 86: 148-155.

51. Veal FC, Bereznicki LRE, Thompson AJ, Peterson GM, Orlikowski C. Subacute Pain as a Predictor of Long-Term Pain Following Orthopedic Surgery An Australian Prospective 12 Month Observational Cohort Study. Medicine 2015; 94.

52. Bellamy N, Buchanan WW, Goldsmith CH, Campbell J, Stitt LW. Validation study of WOMAC: a health status instrument for measuring clinically important patient relevant outcomes to antirheumatic drug therapy in patients with osteoarthritis of the hip or knee. J Rheumatol 1988; 15: 1833-1840.

53. Insall JN, Dorr LD, Scott RD, Scott WN. Rationale of the Knee Society clinical rating system. Clinical Orthopaedics and Related Research 1989: 13-14.

For peer review only - http://bmjopen.bmj.com/site/about/guidelines.xhtml

54. Bafeta A, Dechartres A, Trinquart L, Yavchitz A, Boutron I, Ravaud P. Impact of single centre status on estimates of intervention effects in trials with continuous outcomes: meta-epidemiological study. BMJ 2012; 344: e813.

55. Murray DW, Britton AR, Bulstrode CJK. Loss to follow-up matters. J Bone Joint Surg Br 1997; 79-B: 254-257.

56. Kim J, Lonner JH, Nelson CL, Lotke PA. Response Bias: Effect on Outcomes Evaluation by Mail Surveys after Total Knee Arthroplasty. Journal of Bone and Joint Surgery - Series A 2004; 86: 15-21.

57. Stroup DF, Berlin JA, Morton SC, Olkin I, Williamson GD, Rennie D, et al. Meta-analysis of observational studies in epidemiology: a proposal for reporting. Meta-analysis Of Observational Studies in Epidemiology (MOOSE) group. JAMA 2000; 283: 2008-2012.

58. Dawson J, Fitzpatrick R, Murray D, Carr A. Questionnaire on the perceptions of patients about total knee replacement. The Journal of bone and joint surgery. British volume 1998; 80: 63-69.

59. Lopez-Olivo MA, Landon GC, Siff SJ, Edelstein D, Pak C, Kallen MA, et al. Psychosocial determinants of outcomes in knee replacement. Annals of the rheumatic diseases 2011; 70: 1775-1781.

60. Bellamy N. WOMAC: a 20-year experiential review of a patient-centered self-reported health status questionnaire. J Rheumatol 2002; 29: 2473-2476.

61. Harris K, Dawson J, Doll H, Field RE, Murray DW, Fitzpatrick R, et al. Can pain and function be distinguished in the Oxford Knee Score in a meaningful way? An exploratory and confirmatory factor analysis. Quality of Life Research: An International Journal of Quality of Life Aspects of Treatment, Care & Rehabilitation 2013; 22: 2561-2568.

62. van den Akker-Scheek I, Stevens M, Groothoff JW, Bulstra SK, Zijlstra W. Preoperative or postoperative self-efficacy: which is a better predictor of outcome after total hip or knee arthroplasty? Patient education and counseling 2007; 66: 92-99.

For peer review only - http://bmjopen.bmj.com/site/about/guidelines.xhtml

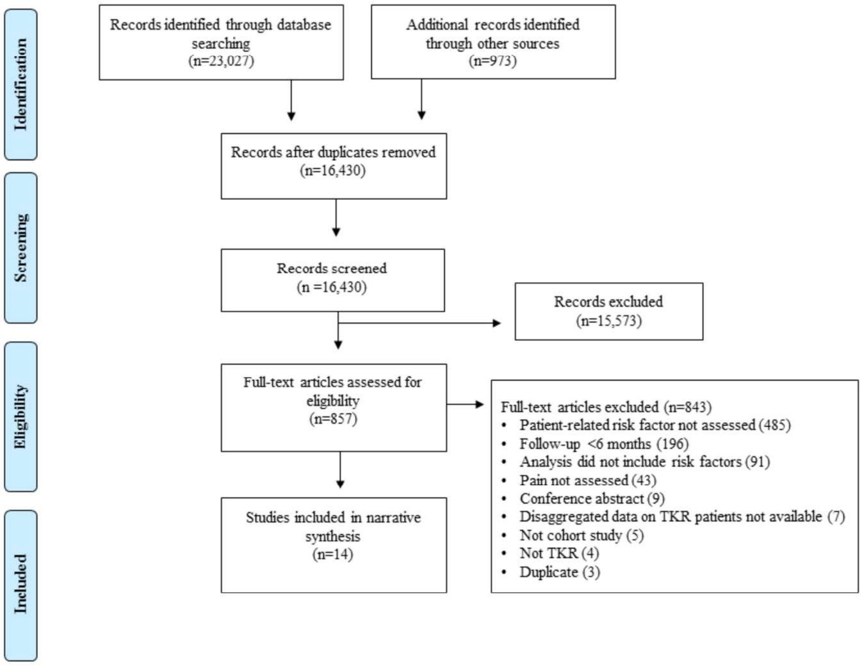

Figure 1: Systematic review flow diagram

181x135mm (300 x 300 DPI)

PRISMA 2009 Checklist

Section/topic	#	Checklist item	Reported on page #
**TITLE**			
Title	1	Identify the report as a systematic review, meta-analysis, or both.	1
**ABSTRACT**			
Structured summary	2	Provide a structured summary including, as applicable: background; objectives; data sources; study eligibility criteria, participants, and interventions; study appraisal and synthesis methods; results; limitations; conclusions and implications of key findings; systematic review registration number.	2
**INTRODUCTION**			
Rationale	3	Describe the rationale for the review in the context of what is already known.	3-4
Objectives	4	Provide an explicit statement of questions being addressed with reference to participants, interventions, comparisons, outcomes, and study design (PICOS).	3-4
**METHODS**			
Protocol and registration	5	Indicate if a review protocol exists, if and where it can be accessed (e.g., Web address), and, if available, provide registration information including registration number.	4
Eligibility criteria	6	Specify study characteristics (e.g., PICOS, length of follow-up) and report characteristics (e.g., years considered, language, publication status) used as criteria for eligibility, giving rationale.	4-5
Information sources	7	Describe all information sources (e.g., databases with dates of coverage, contact with study authors to identify additional studies) in the search and date last searched.	5
Search	8	Present full electronic search strategy for at least one database, including any limits used, such that it could be repeated.	Appendix 3
Study selection	9	State the process for selecting studies (i.e., screening, eligibility, included in systematic review, and, if applicable, included in the meta-analysis).	5-6
Data collection process	10	Describe method of data extraction from reports (e.g., piloted forms, independently, in duplicate) and any processes for obtaining and confirming data from investigators.	5-6
Data items	11	List and define all variables for which data were sought (e.g., PICOS, funding sources) and any assumptions and simplifications made.	5-6
Risk of bias in individual studies	12	Describe methods used for assessing risk of bias of individual studies (including specification of whether this was done at the study or outcome level), and how this information is to be used in any data synthesis.	6
Summary measures	13	State the principal summary measures (e.g., risk ratio, difference in means).	7
Synthesis of results	14	Describe the methods of handling data and combining results of studies, if done, including measures of consistency (e.g., I^2) for each meta-analysis.	7

For peer review only - http://bmjopen.bmj.com/site/about/guidelines.xhtml

PRISMA 2009 Checklist

Section/topic	#	Checklist item	Reported on page #
Risk of bias across studies	15	Specify any assessment of risk of bias that may affect the cumulative evidence (e.g., publication bias, selective reporting within studies).	6
Additional analyses	16	Describe methods of additional analyses (e.g., sensitivity or subgroup analyses, meta-regression), if done, indicating which were pre-specified.	7
**RESULTS**			
Study selection	17	Give numbers of studies screened, assessed for eligibility, and included in the review, with reasons for exclusions at each stage, ideally with a flow diagram.	7, Figure 1
Study characteristics	18	For each study, present characteristics for which data were extracted (e.g., study size, PICOS, follow-up period) and provide the citations.	7-8, Table 1
Risk of bias within studies	19	Present data on risk of bias of each study and, if available, any outcome level assessment (see item 12).	8, Table 2
Results of individual studies	20	For all outcomes considered (benefits or harms), present, for each study: (a) simple summary data for each intervention group (b) effect estimates and confidence intervals, ideally with a forest plot.	Tables 3-5
Synthesis of results	21	Present results of each meta-analysis done, including confidence intervals and measures of consistency.	N/A
Risk of bias across studies	22	Present results of any assessment of risk of bias across studies (see Item 15).	8, Table 2
Additional analysis	23	Give results of additional analyses, if done (e.g., sensitivity or subgroup analyses, meta-regression [see Item 16]).	N/A
**DISCUSSION**			
Summary of evidence	24	Summarize the main findings including the strength of evidence for each main outcome; consider their relevance to key groups (e.g., healthcare providers, users, and policy makers).	11-13
Limitations	25	Discuss limitations at study and outcome level (e.g., risk of bias), and at review-level (e.g., incomplete retrieval of identified research, reporting bias).	11-12
Conclusions	26	Provide a general interpretation of the results in the context of other evidence, and implications for future research.	12-13
**FUNDING**			
Funding	27	Describe sources of funding for the systematic review and other support (e.g., supply of data); role of funders for the systematic review.	14

From: Moher D, Liberati A, Tetzlaff J, Altman DG, The PRISMA Group (2009). Preferred Reporting Items for Systematic Reviews and Meta-Analyses: The PRISMA Statement. PLoS Med 6(7): e1000097. doi:10.1371/journal.pmed1000097

For more information, visit: www.prisma-statement.org.

For peer review only - http://bmjopen.bmj.com/site/about/guidelines.xhtml

PRISMA 2009 Checklist

For peer review only

For peer review only - http://bmjopen.bmj.com/site/about/guidelines.xhtml

Appendix 2: Search terms

MEDLINE (Ovid) (1946 to 17 October 2016)

1 Epidemiologic Studies/

2 exp Case-Control Studies/

3 exp Cohort Studies/

4 Cross-Sectional Studies/

5 (epidemiologic adj (study or studies)).ab,ti.

6 case control.ab,ti.

7 (cohort adj (study or studies)).ab,ti.

8 cross sectional.ab,ti.

9 cohort analy$.ab,ti.

10 (follow up adj (study or studies)).ab,ti.

11 longitudinal.ab,ti.

12 retrospective$.ab,ti.

13 prospective$.ab,ti.

14 (observ$ adj3 (study or studies)).ab,ti.

15 exp clinical study/

16 randomized controlled trial/

17 15 not 16

18 adverse effect?.ab,ti.

19 1 or 2 or 3 or 4 or 5 or 6 or 7 or 8 or 9 or 10 or 11 or 12 or 13 or 14 or 17 or 18

20 Arthroplasty, Replacement, Knee/

21 Knee Prosthesis/

22 (arthoplast$ adj3 knee$).mp. [mp=title, abstract, original title, name of substance word, subject heading word, keyword heading word, protocol supplementary concept word, rare disease supplementary concept word, unique identifier]

23 (knee$ adj3 replac$).mp. [mp=title, abstract, original title, name of substance word, subject heading word, keyword heading word, protocol supplementary concept word, rare disease supplementary concept word, unique identifier]

For peer review only - http://bmjopen.bmj.com/site/about/guidelines.xhtml

24 (knee adj3 implant$).mp. [mp=title, abstract, original title, name of substance word, subject heading word, keyword heading word, protocol supplementary concept word, rare disease supplementary concept word, unique identifier]

25 20 or 21 or 22 or 23 or 24

26 19 and 25

EMBASE (Ovid) (1980 to 17 October 2016)

1 Epidemiologic Studies/

2 exp Case-Control Studies/

3 exp Cohort Studies/

4 Cross-Sectional Studies/

5 (epidemiologic adj (study or studies)).ab,ti.

6 case control.ab,ti.

7 (cohort adj (study or studies)).ab,ti.

8 cross sectional.ab,ti.

9 cohort analy$.ab,ti.

10 (follow up adj (study or studies)).ab,ti.

11 longitudinal.ab,ti.

12 retrospective$.ab,ti.

13 prospective$.ab,ti.

14 (observ$ adj3 (study or studies)).ab,ti.

15 exp clinical study/

16 randomized controlled trial/

17 15 not 16

18 adverse effect?.ab,ti.

19 1 or 2 or 3 or 4 or 5 or 6 or 7 or 8 or 9 or 10 or 11 or 12 or 13 or 14 or 17 or 18

20 Arthroplasty, Replacement, Knee/

21 Knee Prosthesis/

22 (arthoplast$ adj3 knee$).mp. [mp=title, abstract, original title, name of substance word, subject heading word, keyword heading word, protocol supplementary concept word, rare disease supplementary concept word, unique identifier]

For peer review only - http://bmjopen.bmj.com/site/about/guidelines.xhtml

23 (knee$ adj3 replac$).mp. [mp=title, abstract, original title, name of substance word, subject heading word, keyword heading word, protocol supplementary concept word, rare disease supplementary concept word, unique identifier]

24 (knee adj3 implant$).mp. [mp=title, abstract, original title, name of substance word, subject heading word, keyword heading word, protocol supplementary concept word, rare disease supplementary concept word, unique identifier]

25 20 or 21 or 22 or 23 or 24

26 19 and 25

PsycINFO (inception [1806] to 23 March 2016

1. (knee$ adj3 arthoplast$).mp. [mp=title, abstract, heading word, table of contents, key concepts, original title, tests & measures]

2. (knee$ adj3 replac$).mp. [mp=title, abstract, heading word, table of contents, key concepts, original title, tests & measures]

3. (knee$ adj3 surg$).mp. [mp=title, abstract, heading word, table of contents, key concepts, original title, tests & measures]

4. (knee$ adj3 implant$).mp. [mp=title, abstract, heading word, table of contents, key concepts, original title, tests & measures]

5. (knee adj3 prosthe$).mp. [mp=title, abstract, heading word, table of contents, key concepts, original title, tests & measures]

6. 1 or 2 or 3 or 4 or 5

For peer review only

For peer review only - http://bmjopen.bmj.com/site/about/guidelines.xhtml

Appendix 3: Ongoing studies

Ongoing (in recruitment or active) studies identified in a search of ClinicalTrials.gov on the 18th August 2017 using search terms of 'pain', 'observational studies', 'knee replacement', and 'adult, senior'

ClinicalTrials.gov Identifier	Study title	Status	Sponsor	Estimated enrollment	Post-operative risk factor(s)	Pain outcome(s)
NCT01320150	Risk Factors and Mechanisms for Persistent Postsurgical Pain After Total Knee Replacement	Recruiting	Rush University Medical Center	300	Area of secondary mechanical hyperalgesia or hypoalgesia, pain intensity	Numerical Rating Scale at 6 months post-operative
NCT02626533	Persistent Postoperative Pain and Joint Stiffness After Total Knee Arthroplasty Performed for Osteoarthritis	Recruiting	Hospital for Special Surgery, New York	186	Range of motion, pain intensity, KOOS scores, neuropathic pain, time to attainment of inpatient physical therapy goals	Numerical Rating Scale at 6 months post-operative
NCT01390298	Pain and Function After Orthopedic Surgery	Recruiting	Wake Forest University	75	Pain	McGill Pain Questionnaire Short Form at post-operative day 168
NCT02156453	Functional Recovery After Total Knee Arthroplasty	Recruiting	Mahidol University	60	Pain, function	Visual Analogue Scale at 1 year post-operative
NCT02579538	Flexibility of Cognition And Persistent Pain	Ongoing, but not recruiting	Washington University School of Medicine	300	Pain	Pain at 1 year post-operative

For peer review only - http://bmjopen.bmj.com/site/about/guidelines.xhtml
